# Astrocytic β-catenin signaling via TCF7L2 regulates synapse development and social behavior

**DOI:** 10.1038/s41380-023-02281-y

**Published:** 2023-10-05

**Authors:** Lukasz Mateusz Szewczyk, Marcin Andrzej Lipiec, Ewa Liszewska, Ksenia Meyza, Joanna Urban-Ciecko, Ludwika Kondrakiewicz, Anna Goncerzewicz, Kamil Rafalko, Tomasz Grzegorz Krawczyk, Karolina Bogaj, Ilia Davidovich Vainchtein, Hiromi Nakao-Inoue, Alicja Puscian, Ewelina Knapska, Stephan J. Sanders, Tomasz Jan Nowakowski, Anna Victoria Molofsky, Marta Barbara Wisniewska

**Affiliations:** 1 Department of Psychiatry and Behavioral Sciences/Weill Institute for Neurosciences, University of California, San Francisco, San Francisco, CA USA; 2 Laboratory of Molecular Neurobiology, Centre of New Technologies, University of Warsaw, Warsaw, Poland; 3 Laboratory of Emotions Neurobiology, BRAINCITY—Center of Excellence for Neural Plasticity and Brain Disorders, Nencki Institute of Experimental Biology, Polish Academy of Sciences, Warsaw, Poland; 4 Laboratory of Molecular and Cellular Neurobiology, International Institute of Molecular and Cell Biology, Warsaw, Poland; 5 Laboratory of Electrophysiology, Nencki Institute of Experimental Biology, Polish Academy of Sciences, Warsaw, Poland; 6 Reasonfield Lab, Warsaw, Poland; 7 SoftwareMill, Warsaw, Poland; 8 Johnson & Johnson, Neuroscience Therapeutic Area, San Diego, CA USA; 9 Institute of Developmental and Regenerative Medicine, Department of Paediatrics, University of Oxford, Oxford, OX3 7TY UK; 10 New York Genome Center, New York, NY USA; 11 Department of Anatomy, University of California, San Francisco, San Francisco, CA USA; 12 Department of Neurological Surgery, University of California San Francisco, San Francisco, CA USA; 13 The Eli and Edythe Broad Center of Regeneration Medicine and Stem Cell Research, University of California San Francisco, San Francisco, CA USA; 14 Kavli Institute for Fundamental Neuroscience, University of California, San Francisco, San Francisco, CA USA

**Keywords:** Neuroscience, Molecular biology

## Abstract

The Wnt/β-catenin pathway contains multiple high-confidence risk genes that are linked to neurodevelopmental disorders, including autism spectrum disorder. However, its ubiquitous roles across brain cell types and developmental stages have made it challenging to define its impact on neural circuit development and behavior. Here, we show that TCF7L2, which is a key transcriptional effector of the Wnt/β-catenin pathway, plays a cell-autonomous role in postnatal astrocyte maturation and impacts adult social behavior. TCF7L2 was the dominant Wnt effector that was expressed in both mouse and human astrocytes, with a peak during astrocyte maturation. The conditional knockout of *Tcf7l2* in postnatal astrocytes led to an enlargement of astrocytes with defective tiling and gap junction coupling. These mice also exhibited an increase in the number of cortical excitatory and inhibitory synapses and a marked increase in social interaction by adulthood. These data reveal an astrocytic role for developmental Wnt/β-catenin signaling in restricting excitatory synapse numbers and regulating adult social behavior.

## Indroduction

The *CTNNB1* gene, which encodes β-catenin, and *TCF7L2* are high-confidence risk genes that are implicated in psychiatric disorders [1], particularly autism spectrum disorder (ASD) [2–4], characterized by deficits in social interaction and communication. However, these genes are also implicated in global developmental delay [5, 6], underscoring the importance of disentangling potential mechanistic underpinnings of these social and cognitive impacts. Mice that are globally heterozygous for *Tcf7l2* exhibit a decrease in contextual fear learning [7, 8], suggesting that its role in regulating behavior may be accessible in rodent models. However, unknown are which cell types contribute to these potential effects and the ways in which TCF7L2 impacts ASD-relevant behavioral phenotypes, such as social behavior.

The Wnt/β-catenin pathway plays essential and multifaceted roles in brain development that vary by cell type and developmental stage [9–11]. In canonical Wnt signaling, β-catenin translocates to the nucleus where it interacts with its effectors LEF1/TCF proteins to regulate gene expression [12]. TCF7L2 is a Wnt effector with a particularly prominent role in brain development, including promoting the proliferation of radial glia [13], driving oligodendrocyte maturation [14], and regulating the terminal selection of thalamic neurons [15]. *TCF7L2* is expressed in both human and murine glial lineage cells [16, 17], with the highest expression in oligodendrocyte precursor cells and protoplasmic astrocytes [17, 18].

Astrocytes play essential roles in the regulation of neuronal development and function by promoting excitatory synapse formation, regulating neurotransmitter reuptake, and providing metabolic support to neurons [19–22]. Astrocytes are specified from radial glial progenitors beginning in mid-embryogenesis [23], but their development and functional maturation continue throughout postnatal development, coinciding with synapse maturation [24]. During this period, astrocytes exhibit an increase in process complexity and upregulate markers of functional maturity, such as gap junction connexins [25] and potassium channels [26, 27]. TCF7L2 has been implicated in astrocyte specification from neural progenitors [28, 29], but whether it plays ongoing roles in astrocyte lineage and whether it is relevant to roles of astrocyte in neural circuit function are unknown.

The present study identified an astrocyte-specific role for TCF7L2 in the regulation of brain maturation. We established a mouse model to delete *Tcf7l2* from astrocytes after postnatal day 6-7 (P6-7), following the major period of cell proliferation but before completion of the functional maturation of astrocytes. We found that astrocyte-encoded TCF7L2 is required for the morphological, molecular, and functional maturation of astrocytes. Moreover, the lack of TCF7L2 in postnatal astrocytes led to an increase in cortical synapse numbers and synaptic strength. We also found that mice with the astrocyte-specific knockout of *Tcf7l2* exhibited no evident changes in cognition but a marked increase in social behavior. These data reveal a specific role for the β-catenin signaling effector TCF7L2 in restricting the number of excitatory synapses and sociability, with implications for the ways in which human genetic variants in this pathway may affect social behavior in the context of disease.

## Results

### TCF7L2 is a high-confidence ASD risk gene

The β-catenin effector that is encoded by the *TCF7L2* gene has been associated with both developmental delay [5] and ASD [2] through observations of multiple de novo mutations from exome sequencing analyses of large cohorts. The small size of this gene makes this risk score particularly notable because mutation frequencies are proportional to gene length. To explore these data, we mapped 12 reported coding sequence mutations of *TCF7L2*, 10 of which were associated with developmental delay and two were associated with ASD (Extended Data Fig. 1). The majority of the identified variants were in the protein interaction and DNA binding domains (exons 6-14). All were predicted to impact both known protein isoforms of TCF7L2: the full-length (FL TCF7L2) form and a short isoform that lacks the N-terminal β-catenin binding domain and thus acts as a dominant-negative (DN TCF7L2) antagonist of canonical Wnt signaling [30]. Many of these de novo mutations were protein-truncating variants, which are predicted to reduce protein levels, whereas very few truncating variants were observed in the general population [31]. This finding suggests that TCF7L2 haploinsufficiency (i.e., the disruption of one copy of the TCF7L2 gene) contributes to the risk of neurodevelopmental disorders.

### The canonical Wnt pathway effector TCF7L2 is expressed in human astrocytes

**Fig. 1:**
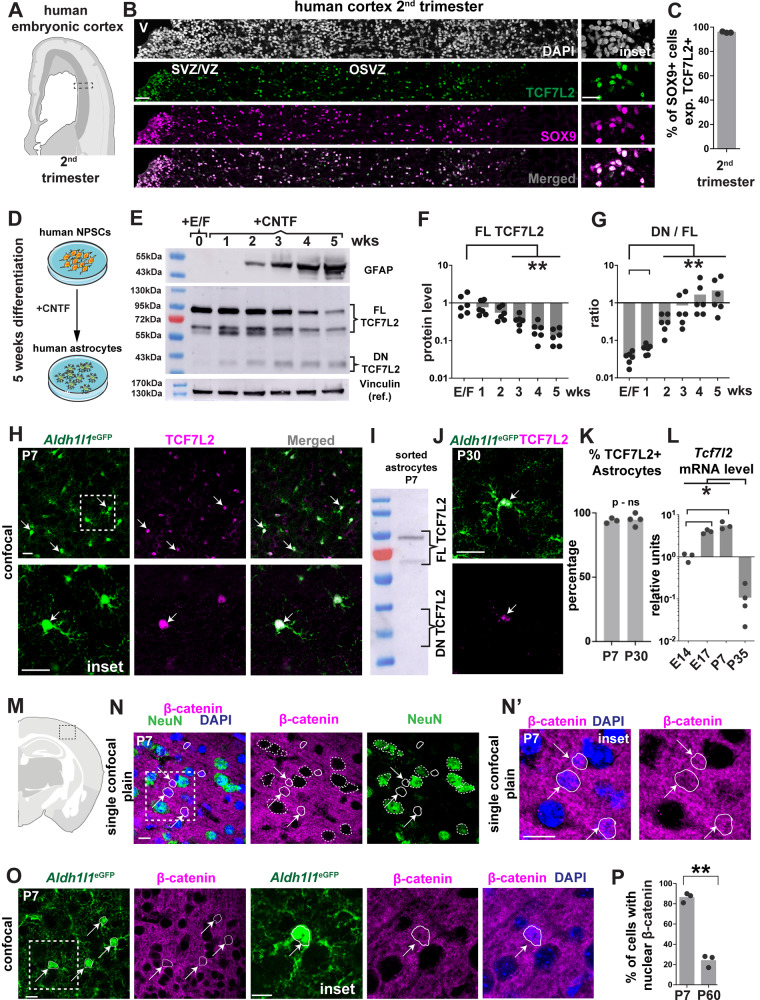
The Wnt effector *Tcf7l2* is expressed in human and rodent astrocyte lineage cells during brain development. **A** Schematic of human cortex, second trimester. The box indicates the region that was used for quantification. **B** Representative image of DAPI (white), TCF7L2+ cells (green), and SOX9+ cells (magenta) in a human cortical section at gestational week 22 (GW22) in the subventricular zone/ventricular zone (SVZ/VZ) and outer subventricular zone (OSVZ). Scale bar = 50 µm (rows 1–4), 30 µm (insets). **C** Percentage of SOX9+ cells in the VZ, SVZ, and OSVZ that expressed TCF7L2 (green) in the second trimester of pregnancy. *n* = 3 biological replicates per age at GW17, GW18, and GW22. Dots represent individual subjects. The data are expressed as the mean. **D** Experimental design of 5 weeks of in vitro CNTF-dependent differentiation of iPSCs-derived human neural progenitor cells (NPSCs) into astrocytes. **E** Representative Western blots of GFAP, TCF7L2 isoforms, and Vinculin (reference) in lysates of differentiated hNPSCs. wks, week; +E/F, fibroblast growth factor (FGF)/epidermal growth factor (EGF) supplementation; CNTF, ciliary neurotropic factor supplementation; FL TCFL2, full-length isoform of TCF7L2 that contained the β-catenin binding domain; DN TCF7L2, dominant-negative isoform of TCF7L2 that lacked β-catenin binding domain. **F** Densitometric analysis of FL TCF7L2 isoforms in differentiated-to-astrocyte hNPSCs normalized to vinculin; (**G**) and ratio of DN TCF7L2 to FL TCF7L2 levels. The data were analyzed using two-tailed Mann–Whitney test. Dots represent individual hNPSC culture. The data are expressed as the mean. Note log scale (**H**) Representative images of *Aldh1l1*^eGFP+^ astrocytes (green) that expressed TCF7L2 protein (magenta) in the somatosensory cortex of mice on postnatal day 7 (P7). Scale bars = 20 µm. **I** Representative Western blot of TCF7L2 isoforms in lysates of sorted astrocytes that were isolated from the cortex of P7 *Aldh1l1*^GFP+^ mice. **J** Representative images of *Aldh1l1*^eGFP+^ astrocytes (green) that expressed TCF7L2 protein (magenta) in the somatosensory cortex of mice on P30. Scale bars = 20 µm. **K** Percentage of *Aldh1l1*^eGFP+^ cells that expressed TCF7L2 in the somatosensory cortex of mice on P7 and P30. Dots represent individual mice. The data are expressed as the mean. The data were analyzed using two-tailed Mann–Whitney test. **L** Expression of *Tcf7l2* by qPCR in *Aldh1l1*^eGFP+^ flow-sorted astrocytes on E14, E17, P7, and P35, normalized to the *Gapdh* housekeeper gene. Dots represent individual mice. The data are expressed as the mean. The data were analyzed using one-way analysis of variance ANOVA with multiple comparisons. **M** Schematic of the region of the somatosensory cortex where quantification was performed. **N, N’** Representative images of Neun+ neurons (green), β-catenin (magenta), and DAPI (blue) in the somatosensory cortex of mice on P7. The white arrow indicates nuclei of non-neuronal cells (white lines) with the nuclear localization of β-catenin. The dashed line indicates nuclei of neurons that lacked the nuclear localization of β-catenin. Scale bars = 10 µm. **O** Representative images of *Aldh1l1*^eGFP+^ astrocytes (green), β-catenin (magenta), and DAPI (blue) in the somatosensory cortex of mice on P7. The white arrow indicates cell soma and nuclei of astrocytes (white lines) with the nuclear localization of β-catenin. Scale bars = 10 µm. **P** Percentage of astrocytes that expressed nuclear localized β-catenin at the indicated ages. Dots represent individual mice. The data are expressed as the mean. The data were analyzed using two-tailed unpaired *t* test with welch’s correction. *p < 0.05, **p < 0.01, ***p < 0.001.

To determine which human brain cells, express TCF7L2, we used bioinformatic and in situ approaches. A dataset of the single-cell RNA sequencing (scRNA-seq) of cortical cells indicated that *TCF7L2* is expressed in oligodendrocyte precursor cells, some neuronal populations, and the astrocyte lineage throughout development. Strikingly, *TCF7L2* maintains its expression in adult human astrocytes [17] (Extended Data Fig. 2A-C). Immunostaining for TCF7L2 in second-trimester embryonic cortex (17-22 weeks of gestation, human tissue from elective pregnancy terminations; Fig. 1A, B, Extended Data Fig. 3A) showed that TCF7L2-positive cells were found throughout the ventricular zone (VZ), subventricular zone (SVZ) and outer subventricular zone (OSVZ) of which ~95% co-labeled with SOX9 [16, 32], a marker of neural progenitors and astrocyte lineage cells (Fig. 1C). We also identified TCF7L2 + /SOX9+ cells in the SVZ in 100-day-old cerebral organoids that were generated from human induced pluripotent stem cells (iPSCs; Extended Data Fig. 3B).

To analyze the ways in which TCF7L2 levels change during human astrocyte differentiation, we quantified TCF7L2 in vitro during 5 weeks of the differentiation of iPSC-derived human neural progenitor stem cells (hNPSCs) into astrocytes in the presence of ciliary neurotrophic factor (CNTF; Fig. 1D). Using Western blot, we first confirmed that neural progenitors differentiate into astrocytes by analyzing glial fibrillary acidic protein (GFAP) levels, which were upregulated as expected (Fig. 1E, upper). Next, we tested expression of the FL TCF7L2 isoform, which declined after 35 days throughout the in vitro maturation process (Fig. 1E, F). Interestingly, we also detected an increase in expression of the DN TCF7L2 isoform (Extended Data Fig. 3C, Fig. 1G). This suggests that during late phases of astrocyte development, canonical β-catenin signaling might be diminished.

### The canonical Wnt pathway effector TCF7L2 is expressed in rodent astrocytes

Next, we tested whether murine astrocytes also express TCF7L2 and other LEF1/TCF family members. Among these β-catenin effectors, only TCF7L2 was detected in P7 cortical astrocytes (Fig. 1H, Fig. 1K - left, Extended Data Fig. 4A) where it was expressed in almost 94% of astrocytic cells. The Western blot analysis of TCF7L2 that was performed on fluorescence-activated cell sorting (FACS)-sorted cells that were isolated from the P7 and P35 cortex of *Aldh1l1*^eGFP^ reporter line showed that murine astrocytes expressed the FL TCF7L2 isoform but not the dominant-negative isoform (Fig. 1I, Extended Data Fig. 4B). At P30, TCF7L2 was still observed in P30 cortical astrocytes but at substantially lower expression levels (Fig. 1J, Fig. 1K, right, Extended Data Fig. 5A, B). In agreement with previous findings [14, 33], we observed the abundant expression of TCF7L2 in a subset of oligodendrocyte lineage cells (Extended Data Fig. 5C, D) and in thalamic neurons (Extended Data Fig. 5E).

To further confirm our findings, we quantified *Tcf7l2* expression at multiple stages of astrocyte lineage maturation [34] using sorted astrocytes from the *Aldh1l1*^eGFP^ reporter line (Extended Data Fig. 6A). We found that *Tcf7l2* mRNA levels peaked in the late embryonic to early postnatal period and decreased with age (Fig. 1L). Parallel experiments that were performed on astrocytes from CNTF differentiated neuroepithelial stem cells (NSCs) also showed a peak and lower levels of FL TCF7L2 with age (Extended Data Fig. 7A-F). In agreement with our in vivo mouse data, we did not observe a DN TCF7L2 isoform in vitro. Altogether, our data demonstrate that TCF7L2 is expressed in both murine and human astrocytes during brain maturation.

### The canonical Wnt pathway is active in cortical astrocytes

Considering that TCF7L2 is a prominent effector of the canonical Wnt pathway, its expression might indicate the activity of its pathway within the astrocyte lineage. To determine whether the Wnt pathway is active in astrocytes, we analyzed its hallmarks, namely the nuclear localization of β-catenin and *Axin2* expression. Immunochemistry showed the high and abundant expression of β-catenin in cell membranes where it plays Wnt-independent roles in cadherin-mediated cell-cell adhesion. However, we also observed nuclear localization within a subset of cortical cells. Staining with the neuronal marker NeuN did not overlap with the nuclear localization of β-catenin, implying that nuclear β-catenin-positive cells belong to glial lineages (Fig. 1M-N**’**). To determine whether nuclear β-catenin-positive cells are astrocytes, we used the *Aldh1l1*^eGFP^ reporter line and quantified the number of ALDH1L1+ cells (Fig. 1O) that expressed β-catenin in the cell nucleus. We observed nuclear β-catenin in 86% of astrocyte nuclei on P7, which declined to 24% of astrocytes by P60 (Fig. 1P, Extended Data Fig. 8A). To further confirm these findings, we quantified the expression of *Axin2*, a downstream marker of Wnt signaling [34, 35], using sorted astrocytes from the *Aldh1l1*^eGFP^ reporter line. As expected, we found that *Axin2* expression increased in the late embryonic to early postnatal period and declined in adult astrocytes (Extended Data Fig. 8B).

Our findings suggest that murine astrocytes exhibit a reduction of the activity of canonical Wnt signaling with age, thereby decreasing the level of its effector TCF7L2. Altogether, these data show that β-catenin signaling is active in astrocytes during late embryonic and postnatal maturation.

### Generation of *Tcf7l2* conditional knockout in postnatal astrocytes

**Fig. 2:**
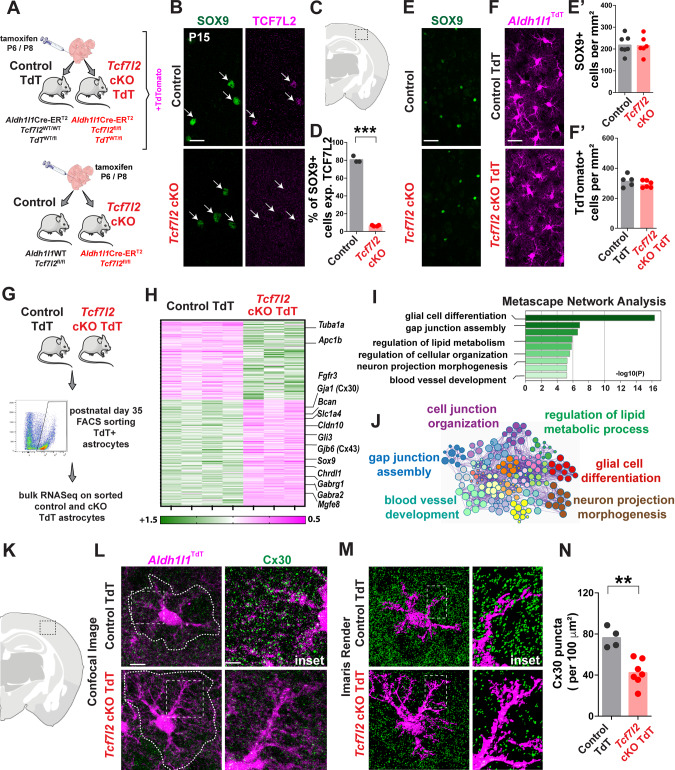
TCF7L2 promotes a gene expression program that is associated with astrocyte functional maturation. **A** Strategy for the tamoxifen-dependent conditional deletion of *Tcf7l2* in postnatal astrocytes using *Aldh1l1*^Cre/ERT2^ mice and a tamoxifen injection on P6 and P8. **B** Representative images of SOX9 (green) and TCF7L2 (magenta) cells in the somatosensory cortex of control and *Tcf7l2* cKO mice. The white arrow indicates astrocytes. Scale bar = 10 µm. **C** Schematic of the region of the somatosensory cortex where quantification was performed. **D** Quantification of the percentage of SOX9 cells with TCF7L2 protein on P15 in control and *Tcf7l2* cKO mice. Dots represent individual mice. The data are expressed as the mean. The data were analyzed using two-tailed unpaired *t* test with welch’s correction. **E, E’** Representative images of SOX9+ (green) cells in the somatosensory cortex of control and *Tcf7l2* cKO. The quantification shows the number of SOX9+ cells per mm^2^ in control and *Tcf7l2* cKO mice. Dots represent individual mice. The data are expressed as the mean. The data were analyzed using two-tailed Mann–Whitney test. Scale bar = 10 µm. **F, F’** Representative images of TdT+ (magenta) astrocytes in the somatosensory cortex of Control TdT and *Tcf7l2* cKO TdT mice. Quantification shows the number of TdT+ astrocytes per mm^2^ in Control TdT and *Tcf7l2* cKO mice. Dots represent individual mice. The data are expressed as the mean. The data were analyzed using two-tailed Mann–Whitney test. Scale bar = 40 µm. **G** Experimental workflow of the isolation of TdT-expressing astrocytes from P35 Control TdT and *Tcf7l2* cKO TdT followed by bulk RNA-seq. **H** Heatmap of the top 275 differentially expressed genes in *Tcf7l2* cKO TdT vs. Control TdT astrocytes in the somatosensory cortex (0.75 >fold change control/cKO >1.25, adjusted *p* < 0.05). Columns indicate individual biological replicates. **I** Top Gene Ontology terms for down**-** and upregulated genes in *Tcf7l2* cKO TdT mice. **J** Metascape visualization of the interactome network that was formed by all 275 differentially expressed genes. **K** Schematic of the region of the somatosensory cortex where Connexin-30 quantification was performed. **L**, **M** Representative images of TdTomato+ astrocytes in the somatosensory cortex of Control TdT and *Tcf7l2* cKO TdT immunostained for Connexin-30 protein (Cx30) (green). Scale bar = 10 µm. The image was rendered three-dimensionally with Imaris software. Scale bar = 2 µm. Note astrocyte fine processes are not visible in this rendering. The dotted line indicates astrocyte domains within which Cx30 puncta were located. **N** Quantification of Cx30 puncta per 100 μm^2^ in the somatosensory cortex of Control TdT and *Tcf7l2* cKO TdT mice on P30. Dots represent individual mice. The data are expressed as the mean. The data were analyzed using two-tailed Mann–Whitney test. *p < 0.05, **p < 0.01, ***p < 0.001.

To specifically examine the role of TCF7L2 in astrocyte maturation, we used *Tcf7l2*^*fl/fl*^ animals with loxP sites that flanked exon 6, the deletion of which causes a frameshift mutation and leads to nonsense-mediated decay. These mice were crossed with *Aldh1l1*Cre-ER^T2^ animals, which carried a transgene with tamoxifen-inducible Cre recombinase under the control of an astrocyte lineage-specific promoter [36] (Fig. 2A, Extended Data Fig. 9A). Tamoxifen (75 mg/kg body weight) was administered on P6 and P8. For flow sorting, we incorporated a cytoplasmic astrocyte TdTomato reporter, *Tcf7l2* conditional knockout (cKO) TdT (*Aldh1l1*Cre-ER^T2^*:Tcf7l2*^*fl/fl*^*:TdTomato*^*WT/fl*^), and a control line *(Aldh1l1*Cre-ER^T2^ *:Tcf7l2*^*WT/WT*^*:TdTomato*^*WT/fl*^ [Control TdT]). In experiments that did not require incorporating a fluorescent reporter, we used a breeding strategy that generated the *Tcf7l2* cKO (*Aldh1l1*Cre-ER^T2^*:Tcf7l2*^*fl/fl*^) genotype and control mice (*Aldh1l1*WT*:Tcf7l2*^*fl/fl*^). This paradigm led to a > 85% reduction of cortical astrocytes that expressed all TCF7L2 isoforms in *Tcf7l2* cKO mice relative to littermate controls (Fig. 2B-D). Importantly, we did not observe a reduction of TCF7L2 levels in other cell types in the brain, such as thalamic neurons (Extended Data Fig. 9B-B”) and oligodendrocyte lineage cells (Extended Data Fig. 9C-C”), which are known to express high levels of TCF7L2. Next, we quantified the number of astrocytes in cortices of *Tcf7l2* cKO and *Tcf7l2* cKO TdT mice and did not observe changes compared with respective control mice (Fig. 2E-E’ and Fig. 2F-F**’**, Extended Data Fig. 10A). We also found that a ratio of neurons to astrocytes did not change (Extended Data Fig. 10B-D), suggesting that overall astrocyte proliferation or the timing of the switch from neurogenesis to gliogenesis was not impacted by TCF7L2 depletion. Thus, this model efficiently and specifically depleted TCF7L2 in astrocytes during postnatal development without impacting the number of astrocytes.

### TCF7L2 promotes a gene expression program that is associated with astrocyte maturation and function

Given the central function of TCF7L2 in the regulation of transcription, we next examined the impact of astrocyte-specific TCF7L2 on gene expression during postnatal development. We used a mouse line that incorporated a cytoplasmic astrocyte TdTomato reporter (*Tcf7l2* cKO TdT) and their littermate control line (Control TdT). We intraperitoneally administered tamoxifen on P6 and P8. Twenty-seven days later, we FACS-sorted TdTomato-positive astrocytes that were isolated from the somatosensory cortex of *Tcf7l2* cKO TdT mice and Control TdT mice and performed bulk RNA-seq (Fig. 2G, Extended Data Fig. 11A, B). The conditional deletion of *Tcf7l2* led to changes in the expression of 275 genes, among which 157 were downregulated and 118 were upregulated (Fig. 2H, Table S1). Gene ontology analysis showed the significant enrichment of networks that are involved in glial cell differentiation, the development of gap junction assembly, and the regulation of neuronal function (Fig. 2I, J). Within these groups, we observed decreases in genes that encode canonical astrocytic regulators of transcription (*Sox9*, *Gli3*, *Id3*, and *Id4*) and genes that encode proteins that are involved in gap junction formation and modulation (*Gjb6*, *Gja1*, and *Cldn10*, which encode Connexin-30, Connexin-43, and Claudine-10, respectively) [37]. We also observed a decrease in the expression of genes that promote synaptic formation and maturation (e.g., *Mgfe8* [Milk fat globule EGF and factor V/VIII domain containing], *Chrdl1* [Chordin like 1], and *Bcan* [Brevican]). Genes that are involved in both excitatory and inhibitory neurotransmission were also altered, including astrocyte-enriched γ-aminobutyric acid (GABA) receptor genes (*Gabrg1* and *Gabra2*) [16] and a D/L-serine transporter [38] (*Slc1a4*). In contrast, there was no clear pattern of upregulated genes in the cKO TdT, although we observed some upregulated genes that were linked to cytoskeleton and actin filaments (*Tuba1a* and *Arpc1b*). Notably, some of the differentially expressed genes (*Id2*, *Sox9*, *Gjb6*, and *Gja1*) are known target genes of Wnt/β-catenin signaling, confirming that *Tcf7l2* deletion effectively lowered activity of the canonical Wnt pathway in astrocytes.

We validated our RNA-seq data at the protein levels by quantifying the expression of astrocytic Connexins (Connexin-30 and Connexin-43) using immunohistochemistry. We observed a significant decrease in the number of Connexin-30 puncta (Fig. 2K–N) and Connexin-43 puncta (Extended Data Fig. 11C–E).

In summary, our findings showed that transcriptional regulation by TCF7L2 promotes an astrocytic program that is associated with their maturation and neuronal support functions, including gap junction coupling and synapse formation.

### Astrocytic TCF7L2 regulates the tiling of astrocytes and gap junction coupling

**Fig. 3:**
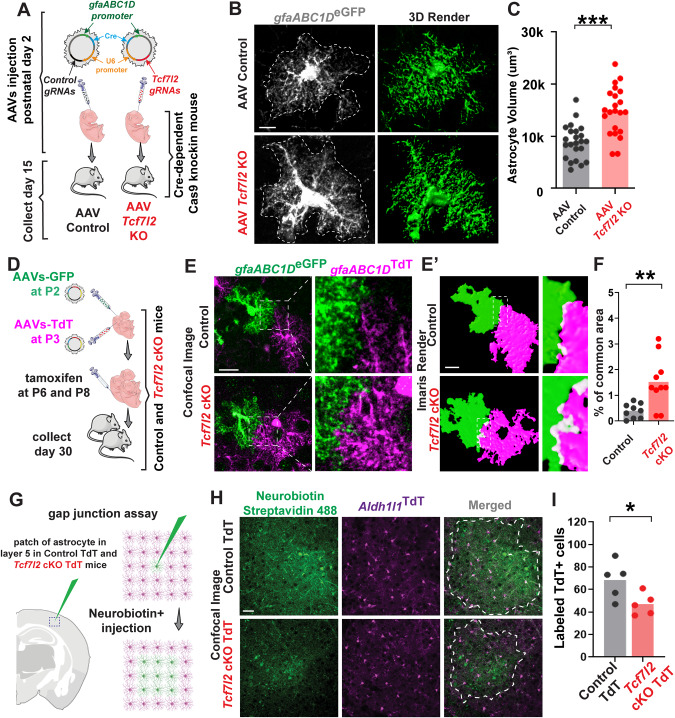
TCF7L2 regulates astrocyte tiling and gap junction coupling. **A** Schematic of the in vivo adeno-associated (AAV)-mediated deletion of *Tcf7l2* in postnatal astrocytes using Cre-dependent Cas9 knockin mice (B6J.129(B6N)-Gt(ROSA)26Sor^tm1(CAG-cas9*-EGFP)Fezh^/J). **B** Representative confocal images of GFP+ astrocytes in the somatosensory cortex of AAV Control and AAV *Tcf7l2* KO mice (left) and their three-dimensional Imaris renders (right). Scale bar = 10 µm. **C** Quantification of the volume of GFP+ astrocytes in the somatosensory cortex of AAV Control and AAV *Tcf7l2* KO. Three-dimensional models of GFP astrocytes were computed from three-dimensional images (confocal z-stacks) by the segmentation function. Calculation of the volume of individual astrocytes was performed in Imaris based on three-dimensional renders of GPF astrocytes. *n* = 5 mice/group. Dots represent individual astrocytes (23 AAV control astrocytes, 22 AAV *Tcf7l2* KO astrocytes). The data are expressed as the mean. The data were analyzed using two-tailed Mann–Whitney test. **D** Schematic of the in vivo microinjection of AAVs that expressed GFP or TdTomato fluorescent protein under the astrocyte-specific *gfaABC1D* promoter on P2 and P3 in control *vs*. *Tcf7l2* cKO pups followed by a tamoxifen injection on P6 and P8. Representative images of GFP+ and TdT+ astrocytes in the somatosensory cortex of control or *Tcf7l2* cKO (**E**) and their three-dimensional Imaris renders (**E’**). Scale bar = 20 µm. **F** Quantification of the common occupied area by two neighboring astrocytes (either TdTomato or GFP) in control and *Tcf7l2* cKO mice. The data were analyzed using two-tailed Mann–Whitney test. **G** Schematic of gap junction coupling assay. Neurobiotin Plus was injected in a single TdTomato+ astrocyte (magenta) in acute cortical slices for 30 min. **H** Representative images of cortical slices that were stained with Streptavidin 488 (green) to detect Neurobiotin Plus-labeled astrocytes in Control TdT and *Tcf7l2* cKO TdT mice on P35. Scale bar = 20 µm. **I** Quantification of Neurobiotin Plus–Streptavidin 488-labeled TdT+ astrocytes in Control TdT and *Tcf7l2* cKO TdT mice. Dots represent individual slices. The data were analyzed using two-tailed unpaired *t* test with welch’s correction The data are expressed as the mean. *p < 0.05, **p < 0.01, ***p < 0.001.

Astrocytes mature morphologically during postnatal cortical development, becoming larger and more ramified during the first 30 postnatal days [24, 39]. To determine the cell-autonomous impact of TCF7L2 on astrocyte morphology, we used the adeno-associated virus (AAV)-mediated delivery of CRISPR/Cas9 to delete *Tcf7l2* (Fig. 3A). On P2, we intraventricularly injected AAVs to express *Cre* under the astrocyte-specific promoter gfaABC1D and two guide RNAs under the U6 promoter (Extended Data Fig. 12A). The efficiency of *Tcf7l2*-targeted guide RNA (gRNA) and a control LacZ-targeted gRNA was validated in vitro and in vivo (Extended Data Fig. 12B, C). Given that the size of astrocytes increases with maturation, we were surprised to find that astrocytes in *Tcf7l2* gRNA-injected mice had a significantly larger volume relative to controls on P15 (Fig. 3B, C).

Maturing astrocytes, in addition to increasing in volume, exhibit tiling in the brain establishing independent and non-overlapping domains. The number of astrocytes in *Tcf7l2* cKO mice was equal to the number of astrocytes in control animals. We sought to determine whether this increase might result in a failure to properly tile, causing astrocytes to invade adjacent astrocyte domains. To test this hypothesis, we used AAVs to sparsely label cortical astrocytes with either green fluorescent protein (GFP; AAVs-GFP) or TdTomato (AAVs-TdT) under the gfaABC1D promoter (Fig. 3D). On P2, we intraventricularly injected AAVs-GFP, followed by AAVs-TdT injections 24 h later. On P6 and P8, we administered tamoxifen. On P30, we quantified astrocyte overlap in neighboring cells of two different colors in control and *Tcf7l2* cKO mice (Fig. 3E, E'). We observed significantly larger overlap in *Tcf7l2* cKO astrocytes compared with control cells (Fig. 3F), consistent with a defect in tiling.

Proper astrocyte tiling is critical for nervous system function, including such processes as synaptic transmission, plasticity, and neuronal excitability [40, 41]. These processes are synchronized across astrocyte territories via gap junction coupling. To test whether the observed molecular and morphological alterations corresponded to changes in astrocytic gap junction coupling, we injected Neurobiotin Plus dye in individual astrocytes in the layer 5 somatosensory cortex in acute slices from P30 Control TdT and *Tcf7l2* cKO TdT mice (Fig. 3G, H) and examined the spreading of dye via gap junctions of coupled astrocytes. We found a significant reduction of dye spread among astrocytes in cKO mice (Fig. 3I). These data indicate impairments in astrocytic gap junction function or assembly in *Tcf7l2* cKO mice.

### Astrocytic TCF7L2 regulates neuronal synapse development and function

**Fig. 4:**
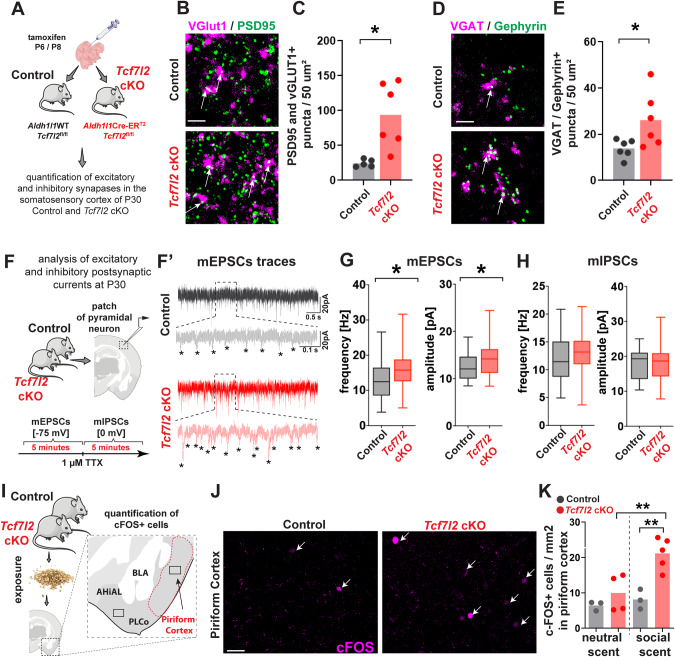
Astrocytic TCF7L2 restricts excitatory synapse numbers and synaptic strength. **A** Strategy for the quantification of excitatory and inhibitory synapses in the somatosensory cortex of P30 control and *Tcf7l2* cKO mice. **B** Representative images of excitatory synapses (VGLUT1, magenta; PSD95, green) Scale bar = 1 µm. **C** Quantification of average synaptic co-localized puncta in layer 5 of somatosensory cortex of control and *Tcf7l2* cKO mice. Dots represent individual mice. The data were analyzed using two-tailed Mann–Whitney test. The data are expressed as the mean. **D** Representative images of inhibitory synapses (VGAT, magenta; Gepherin, green) Scale bar = 1 µm **E** Quantification of average synaptic co-localized puncta in layer 5 of the somatosensory cortex of control and *Tcf7l2* cKO mice. Dots represent individual mice. The data were analyzed using two-tailed Mann–Whitney test. The data are expressed as the mean. **F** Schematic of ex vivo whole-cell patch-clamp recordings of miniature excitatory and inhibitory postsynaptic currents of neurons in layer 5 of the somatosensory cortex in control and *Tcf7l2* cKO mice. **F’** traces from pyramidal neurons in acute cortical slices in P30 control (upper) and *Tcf7l2* cKO (lower) mice. **G** Average neuron mEPSC amplitude (left) and frequency (right) in control and *Tcf7l2* cKO mice. The data are expressed as the mean (*n* = 25 control neurons from 4 control mice; *n* = 44 cKO neurons from 8 Tcf7l2 cKO mice). The data were analyzed using two-tailed Mann–Whitney test. **H** Average neuron mIPSC amplitude (left) and frequency (right) in control and *Tcf7l2* cKO mice. The data are expressed as the mean *(n* = 25 control neurons from 4 control mice; *n* = 44 cKO neurons from 8 *Tcf7l2* cKO mice). The data were analyzed using two-tailed Mann–Whitney test. **I** Schematic of strategy for the quantification of excitability in response to social scent. Control and *Tcf7l2* cKO mice were exposed to a neutral scent (fresh unused bedding) or social scent (used bedding from unfamiliar subjects) for 90 min. The piriform cortex is indicated by red outlines. Outlines indicate the region within which c-FOS+ neurons were quantified. BLA Basolateral Amygdala, PLCo Posterolateral Cortical Amygdala, AHiAL amygdalohippocampal area. **J** Representative images of c-FOS staining in the piriform cortex in control and *Tcf7l2* cKO mice. Scale bar = 40 µm. **K** Quantification of the number of c-FOS+ neurons in the piriform cortex in control and *Tcf7l2* cKO mice after exposure to the neutral or social scent. Dots represent individual mice. The data are expressed as the mean. The data were analyzed using one-way analysis of variance ANOVA with multiple comparisons. *p < 0.05, **p < 0.01, ***p < 0.001.

Astrocytes play critical roles in the development and function of both excitatory and inhibitory synapses. Decreases in the expression of genes that promote synaptic maturation and formation (e.g., *Mgfe8*, *Chrdl1*, and *Bcan*) implicate astrocytic TCF7L2 in cortical synaptogenesis. To further test this hypothesis, we injected tamoxifen on P6 and P8 in *Tcf7l2* cKO mice and their littermate controls (Fig. 4A) and quantified synapse density in layer 5 somatosensory cortex neurons in 30-day-old animals. We measured co-localization of the pre- and postsynaptic markers vesicular glutamate transporter 1 (VGLUT1)/postsynaptic density 95 (PSD95; intracortical/excitatory) and vesicular GABA transporter (VGAT)/gephyrin (inhibitory) [24, 42]. We observed a significant increase in excitatory synaptic puncta (Fig. 4B, C) and a more modest increase in inhibitory synapses in *Tcf7l2* cKO mice (Fig. 4D, E).

Next, to test functional properties of layer 5 somatosensory cortex pyramidal neurons of ‘*Tcf7l2* cKO mice’, we performed whole-cell patch-clamp recordings of miniature excitatory and inhibitory postsynaptic currents (mEPSCs and mIPSCs, respectively; Fig. 4F, F**’**). In P30 mice, we observed an increase in the frequency of mEPSCs in *Tcf7l2* cKO neurons compared with controls (Fig. 4G, left), consistent with the results of the synaptic quantifications. We also observed an increase in mEPSC amplitude, indicating an increase in synaptic strength (Fig. 4G, right). We also noticed a trend toward an increase in the frequency of mIPSCs, but these changes were not statistically significant (Fig. 4H). Thus, astrocyte dysfunction that was induced by *Tcf7l2* knockout resulted in an increase in the number and strength of cortical excitatory synapses.

Finally, to test whether the increase in excitatory activity in *Tcf7l2* cKO neurons translates to behavioral phenotypes, we exposed control and *Tcf7l2* cKO mice to a neutral scent (fresh bedding) or social scent (used bedding from unfamiliar subjects, same sex) to evoke neural activity in the olfactory cortex and quantified neuronal activation by staining with the immediate early gene c-FOS [43] (Fig. 4I). Control animals exhibited a trend toward a higher number of c-FOS-positive cells after exposure to the social scent compared with a neutral scent. *Tcf7l2* cKO animals exhibited a significant and much more robust increase in c-FOS-positive neurons in the piriform cortex (2.7-fold *vs*. control mice; Fig. 4J, K). This finding suggests that neuronal activation is regulated by astrocytes in a TCF7L2-dependent matter. Altogether, these findings show that astrocytic TCF7L2 is critical for cortical synapse development and function and thus could impact behavior.

### Astrocyte-specific postnatal knockout of *Tcf7l2* leads to hypersociability in adulthood

Synaptic dysfunction underlies the pathophysiology of ASD, which is primarily characterized by social and cognitive deficits. Given strong genetic evidence that implicates *Tcf7l2* in both ASD and intellectual disability and our present demonstration of its role in synaptic development, we next investigated whether astrocytic *Tcf7l2* impacts social and cognitive behaviors.

**Fig. 5:**
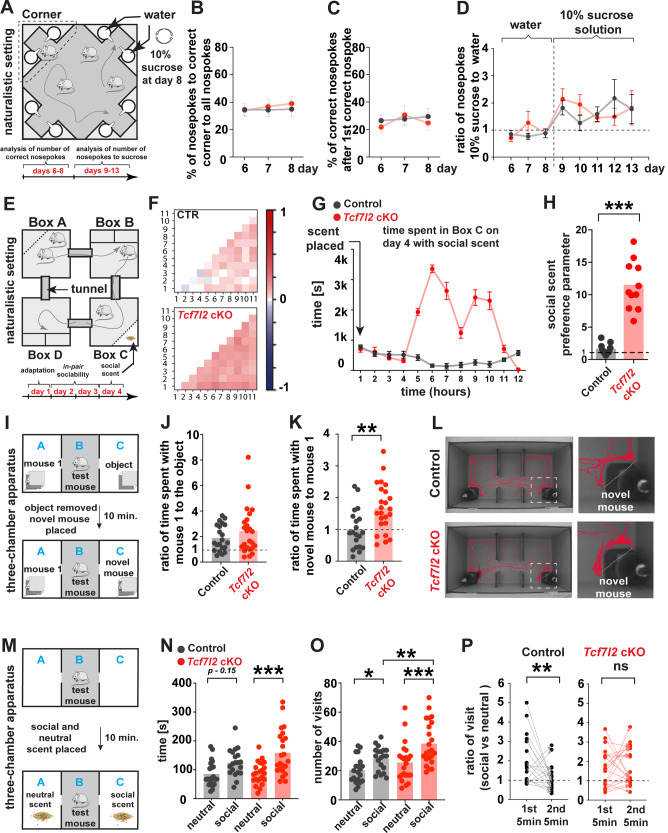
Astrocyte-specific deletion of *Tcf7l2* leads to an increase in sociability. **A** Schematic of naturalistic setting that was used to analyze learning and cognition. The setting was composed of a main arena (housing compartment and four corners). Each corner was composed of two chambers (left and right) that offered access to water. **B** Quantification of correct nosepokes relative to all nosepokes in control and *Tcf7l2* cKO mice during days 6–8 of the experiment. *n* = 12 control, *n* = 13 *Tcf7l2* cKO. The data were analyzed using two-tailed Mann–Whitney test. **C** Quantification of correct nosepokes after the first correct nosepoke in control and *Tcf7l2* cKO mice during days 6-8 of the experiment. The data were analyzed using two-tailed Mann–Whitney test. **D** Ratio of the number of nosepokes to the chamber with 10% sucrose solution to the number of nosepokes to the chamber with tap water. The data were analyzed using two-tailed Mann–Whitney test. **E** Schematic of naturalistic setting that was used for the social behavior analysis. Boxes B and D were housing compartments that offered access to food and water *ad libitum* and provided shelter. Boxes A and C contained no food or water and were equipped with an impassable, transparent, and perforated partition behind which an olfactory stimulus (neutral or social scent) was presented. The boxes were connected by tunnels that were equipped with antennas to detect movements between boxes. The timeline shows the testing phase of habituation (day 1), sociability testing (days 2–3), and exposure to social scent (day 4). **F** Density plot matrices that show sociability in control and *Tcf7l2* cKO mice on day 3 of the experiment, quantified as the time spent together between two mice in a box above the time that would be expected by random exploration or by chance (see methods for full calculation parameters). Each small square in the matrix represents one pair of mice, color coded along a normalized sociability parameter that ranges from –1 to +1. Values from 0 to +1 represent net positive social behavior. Values from 0 to –1 indicate relative avoidance between that pair. *n* = 11 mice/group. **G** Quantification of time spent by control and *Tcf7l2* cKO mice near the social scent (Box C), binned in 1-h intervals (bin). The data are expressed that mean ± SEM. **H** Social scent preference parameter (total time spent near the social scent on day 4). Dots represent individual mice. The data are expressed as the mean. The data were analyzed using two-tailed Mann–Whitney test. **I** Schematic of social novelty assay in three-chamber apparatus. A, B, and C indicate compartments. Boxes A and C contained grid enclosures where mouse 1(stranger 1 mouse), an inanimate object (object), or a novel mouse (stranger mouse 2 - social novelty) were placed. **J** Ratio of spent time by control and *Tcf7l2* cKO mice in close proximity to mouse 1 to time spent in close proximity to the inanimate object. Dots represent individual mice (males and females). The data are expressed as the mean. The data were analyzed using two-tailed Mann–Whitney test. **K** Ratio of spent time by control and *Tcf7l2* cKO mice in close proximity to mouse 1 to time spent in close proximity to novel mouse (stranger mouse 2 - social novelty). Dots represent individual mice (males and females). The data are expressed as the mean. The data were analyzed using two-tailed Mann–Whitney test. **L** Representative traces of control and *Tcf7l2* cKO mice traveling in three-chamber apparatus during exposure to the novel mouse. **M** Schematic of social scent assay in three-chamber arena. A social scent was placed in Box A, and a neutral scent was placed in Box C. **N** Time spent by control and *Tcf7l2* cKO mice in close proximity to either the neutral or social scent. Dots represent individual mice (males and females). The data were analyzed using one-way analysis of variance ANOVA with multiple comparisons. **O** Number of visits by control and *Tcf7l2* cKO mice to either the neutral or social scent. Dots represent individual mice (males and females). The data were analyzed using one-way analysis of variance ANOVA with multiple comparisons. **P** Ratio of visits by control (left) and *Tcf7l2* cKO (right) to social scent during the first and second 5 min of the assay to the visits near the neutral scent. The entire assay lasted 10 min. Dots represent individual mice (males and females). The data were analyzed using two-tailed Mann–Whitney test. *p < 0.05, **p < 0.01, ***p < 0.001.

To assess cognition, we used a naturalistic paradigm (Fig. 5A). The testing arena contained corners that could dispense sweetened or unsweetened water in response to nosepokes. Because of the lower level of aggression in females compared with males, we tested a mouse cohort that was composed only of females in that naturalistic setting (control: *n* = 12, *Tcf7l2* cKO: *n* = 13). After adaptation and habituation to the setting, the mice learned to associate a particular corner with the sweetened water reward, and this place learning was quantified by measuring the number of nosepokes. We observed no changes in learning or memory between control and *Tcf7l2* cKO mice (Fig. 5B-D, Extended Data Fig. 13), suggesting that *Tcf7l2* deletion in astrocytes does not impact cognition.

To observe social behavior in *Tcf7l2* cKO mice compared with their littermate controls, we used a naturalistic setting that was recently developed and tested in mouse models of autism [44] (Fig. 5E**, schematic**). Female cohorts (control: *n* = 11, *Tcf7l2* cKO: *n* = 11 from the same groups as above) were placed in a testing arena that consisted of four chambers that were connected by tunnels. The mice could be individually identified by an implanted transponder, and their movements between chambers were tracked. After 1 day of habituation to the arena (Fig. 5E**, axis**), we measured social interactions between all pairs of mice on days 2 and 3. We quantified the *in-pair sociability parameter* (i.e., the amount of time each pair of mice spent together, a metric that was previously shown to decrease in genetic models of ASD [45]). *Tcf7l2* cKO mice exhibited a two-fold increase in sociability compared with control mice throughout the testing period (Fig. 5F, Extended Data Fig. 14A**[day 3]**, Extended Data Fig. 14B, C**[day 2]**), with no changes in locomotor activity (Extended Data Fig. 14D).

As an independent metric of social preference in this naturalistic setting, we quantified interest in a neutral or social scent on the last day of testing. On day 4 (Fig. 5G), we placed a social scent into Box C and a neutral scent into Box A. *Tcf7l2* cKO mice spent significantly more time in the box with the social scent compared with control mice (Fig. 5H). No box preference was observed before placing the odor cues (Extended Data Fig. 14E).

To independently validate this apparent increase in social preference using a classic and standardized behavioral test, we assessed social behavior using a three-chamber apparatus. We collected new cohorts of control and cKO mice (control: *n* = 12 females, *n* = 9 males; *Tcf7l2* cKO: *n* = 12 females, *n* = 11 males). After habituation to the three-chamber apparatus, each mouse was individually placed in the center of a three-chamber arena. The mice had access to a mouse under a wire cup in one chamber or a wooden block (inanimate object) in the other chamber (Fig. 5I, Extended Data Fig. 15A). After 10 min, the object was removed and replaced with a novel mouse. Both control and *Tcf7l2* cKO mice preferred the mouse over the object (Fig. 5J**[ratio** > **1]**, Extended Data Fig. 15B), indicating normal social preference. However, *Tcf7l2* cKO mice exhibited significantly higher preference for interacting with a novel mouse compared with their littermate controls (Fig. 5K, L, Extended Data Fig. 15C).

Finally, we used a similar standardized paradigm to assess the response to the social scent (Fig. 5M). All mice were again habituated to the apparatus (Extended Data Fig. 16A). Each mouse was next placed in the center and then introduced to a social scent in one chamber and a neutral scent in the other chamber. *Tcf7l2* cKO mice spent more time around the social scent (Fig. 5N) and visited the social scent area significantly more times (Fig. 5O) than their littermate controls. Interestingly, the preference of control mice for the social scent decreased over the 10-min testing window (Fig. 5P, **left**), whereas *Tcf7l2* cKO mice maintained interest in social stimuli during the entire testing period (Fig. 5P, **right**, Extended Data Fig. 16B, C). No changes between cKO males and cKO females in approach to the social scent were observed, suggesting that the TCF7L2-mediated regulation of social behavior is not sex-specific (Extended Data Fig. 16D, E). Collectively, our studies indicate that astrocytic TCF7L2 preferentially impacts male and female social behavior without substantially impacting cognition or learning.

## Discussion

In the present study, we identified the requirement for the β-catenin effector TCF7L2 in regulating astrocyte maturation, neuronal and synapse function, and social behavior. Our study specifically focused on the postnatal impact of the β-catenin/TCF7L2 axis using temporally controlled conditional deletion of the *Tcf7l2* gene to bypass effects of Wnt/β-catenin signaling on early astrogenesis. Our findings provide evidence for the roles of TCF7L2 in promoting astrocyte maturation and demonstrate that the Wnt pathway can regulate synaptic function and influence social behavior via astrocyte-dependent mechanisms.

The postnatal knockout of *Tcf7l2* in astrocytes influenced the expression of genes that encode other developmentally coordinated astrocytic transcription factors (e.g., *Sox9* and *Gli3*). Therefore, TCF7L2 can regulate gene expression directly, indirectly, and in cooperation with other transcription factors. Regardless of whether TCF7L2 alone or in cooperation with other transcription factors is responsible for these changes, we propose that TCF7L2 plays unique roles at different stages of astrocyte lineage development [1], which has also been observed with other transcriptional regulators, such as nuclear factor 1 A-type (NFIA**)** [46].

A mechanistic understanding of the function of transcription factors in physiological phenomena is complicated by their pleiotropic roles, which also applies to the role of TCF7L2 in astrocyte development. Therefore, it is difficult to conclusively state whether different phenotypes that are observed in astrocytes in *Tcf7l2* cKO mice (e.g., impairments in tiling or gap junction dysfunction) are independent or result from each other. Similarly, unclear is whether the aforementioned phenotypes cause an increase in synapse number and function or whether synaptic impairments in the *Tcf7l2* cKO occur independently of them. A recent study indicated such a possibility, in which the dysregulation of Connexin-30 may impact synaptic activity [47]. However, further studies are required to find a direct link between the observed *Tcf7l2* cKO phenotypes and precise molecular mechanisms by which TCF7L2 governs these processes.

Our study highlights the importance of considering cell-type specific effects when evaluating numerous roles of canonical Wnt signaling in synapse maturation, stability, and strength [48]. The precise molecular mechanism by which synaptic function in the somatosensory cortex increased in mice that lacked astrocytic TCF7L2 needs detailed investigation. Nonetheless, our findings show the direct involvement of TCF7L2 in the regulation of genes that encode proteins that are involved in the elimination of synapses (e.g., *Mfge8*, *Chrdl1 Bcan* and *Hgf*). Considering that astrocytes are the major source of secreted MFGE8 protein in the brain [49] and that MFGE8 mediates the phagocytic removal of synapses by microglia [50], its deficiency could decrease synaptic pruning, thereby increasing the number of synapses in *Tcf7l2* cKO mice. We do not know whether the decrease in MFGE8 is caused by developmental impairments in astrocytes in *Tcf7l2* cKO mice (e.g., immature astrocytes release an insufficient level of MFGE8). One possibility is that TCF7L2 regulates expression of the *Mfge8* gene only in a time-specific manner, such as during the period of synapse pruning. Overall, our data strongly suggest the existence of TCF7L2- and astrocyte-dependent mechanisms that contribute to synapse development and function.

Autism spectrum disorder is usually described as a neuronal disorder. For example, pathologically high synapse density appears to be a common feature of ASD [51, 52]. However, recent data highlight abnormalities in human glial cells, such as astrocytes and microglia [17]. For example, protoplasmic astrocytes that were isolated from postmortem ASD brains exhibited molecular and functional alterations. This implies that cell lineages that support neuronal development, maturation, and function, in addition to impairments in neuronal cells, may contribute to synaptic dysfunction in ASD. Our findings corroborate this possibility by showing that impairments in astrocyte maturation are a cause of synaptic dysfunction and social deficits. Changes in the expression of astrocytic transcription factors and an increase in the number of cortical synapses resemble irregularities that are observed in ASD patients [51, 52]. Similarities at the cellular, molecular, and physiological levels and alterations of social interactions make our model a promising and valuable tool for further research on the pathogenesis of ASD.

Alterations of sociability in *Tcf7l2* cKO mice suggest that astrocytic impairments in ASD patients with de novo mutations of the *TCF7L2* gene might contribute to development of the disorder. However, hypersociability in *Tcf7l2* cKO mice is unexpected, considering that lower social motivation is considered a core symptom of ASD. The opposite phenotype of *Tcf7l2* cKO mice cannot be explained by the opposite effect of mutations because the majority of the aforementioned mutations of the human gene and our mouse model cause a STOP codon gain. However, we should not oversimplify the interpretation of social behavior in *Tcf7l2* cKO mice. First, social preference test results cannot be treated as a simple equivalent to social dysfunction in humans. Second, the expression of TCF7L2 in other cell types potentially alters the ultimate phenotype in humans. Finally, our model is not the first putative model of ASD that presents with an increase in social interest. A similar loss of social inhibition was observed in mice with knockout of the PSD95-encoding gene [53–55], the disruption of which was identified as a genetic risk factor for ASD [56]. This is consistent with observations in humans that neurodevelopmental disorders manifest as a spectrum of conditions, and patients can even exhibit apparently opposite behaviors. For example, excessive social behavior is a characteristic of Williams syndrome, which is often depicted as anti-ASD, even though both disorders share social cues blindness and many brain pathologies [57, 58]. Furthermore, ASD phenotypes are, in fact, prevalent in this disorder [59–61].

We conclude that alterations of sociability in our mouse model suggest that astrocytic impairments in ASD patients with de novo mutations of the TCF7L2 gene might contribute to the development of ASD. This raises the intriguing possibility that postnatal astrocytes could be targeted to regulate social behavior.

## Material and methods

### Mice

We used the C57BL/6NTac-Tcf7l2^tm1a^(EUCOMM)Wtsi/WtsiIeg (*Tcf7l2*^*tm1a*^) mouse strain [62] with a trap cassette that was upstream of critical exon 6 of the *Tcf7l2* gene. Mice that were homozygous for the *Tcf7l2*^*tm1a*^ allele have a total knockout of the gene. To generate the *Aldh1l1*Cre-ER^T2^*:Tcf7l2*^*fl/fl*^ strain, in which *Tcf7l2* knockout is induced in astrocytes after the administration of tamoxifen (Merck Life Science, catalog no. T5648-1G, 75 mg/kg body weight), *Tcf7l2*^tm1a/+^ animals were first crossed with flippase-expressing mice (ROSA26:FLPe knock-in strain; Jackson Laboratory, catalog no. 009086) and then with B6;FVB-Tg(Aldh1l1-cre/ERT2)1Khakh/J mice (Jackson Laboratory, catalog no. 029655) that express Cre recombinase from the *Aldh1l1* promoter. *Aldh1l1*WT*:Tcf7l2*^*fl/fl*^ animals were used as controls. To generate the *Aldh1l1*Cre-ER^T2^*:Tcf7l2*^*fl/fl*^*:tdTomato*^WT/fl^ reporter strain, *Aldh1l1*Cre-ER^T2^*:Tcf7l2*^*fl/fl*^ mice were crossed with homozygous Ai9(RCL-tdT) mice (B6.Cg-Gt[ROSA]26Sortm9[CAG-tdTomato]Hze/J; Jackson Laboratory, catalog no. 007909). *Aldh1l1*^eGFP^ transgenic mice (Mutant Mouse Resource and Research Centers, catalog no. 036071-UCD) encode eGFP under control of the *Aldh1l1* promoter. B6J.129(B6N)-Gt(ROSA)26Sortm1(CAG-cas9*-EGFP)Fezh/J transgenic mice (Jackson Laboratory, catalog no. 026175) encode bicistronic Cas9 and the EGFP cassette, the expression of which that is under control of the CAG promoter is induced by Cre-mediated STOP cassette removal. For the experimental procedures, all mice were selected by PCR-based genotyping: *Tcf7l2tm1a* and *Tcf7l2fl* alleles (tcf_F, GGAGAGAGACGGGGTTTGTG; tcf_R, CCCACCTTTGAATGGGAGAC; floxed_PNF, ATCCGGGGGTACCGCGTCGAG; Tm1c_R, CCGCCTACTGCGACTATAGAGA), *Aldh1l1*_Cre allele (31091, CAACAGGTGCCTTCCA; 30308, GGCAAACGGACAGAAGCA), tdTomato (oIMR9105, CTGTTCCTGTACGGCATGG), WPRE (oIMR9103, GGCATTAAAGCAGCGTATC).

All animals were housed under standard laboratory conditions (21°C ± 2°C, 60% ± 10% humidity, and 12 h/12 h light/dark cycle) with food and water provided *ad libitum*. All of the experimental procedures were conducted in compliance with current normative standards of the European Community (86/609/EEC) and Polish government (Dz.U. 2015 poz. 266). All of the protocols for animal use were approved by the Polish Local Ethical Committee No. 1 in Warsaw. Animal use was controlled by the institutional advisory board for animal welfare at the Center of New Technologies.

### Neuroepithelial Stem Cells culture (NSCs)

Neuroepithelial stem cell cultures were set up according to the protocol of Hazel and Muller, 2001 with minor modifications [63]. Briefly, cortices from 3-4 mouse embryos on day 14 of gestation (embryonic day 14.5 [E14.5]) were dissected, transferred to ice-cold Hank’s Balanced Salt Solution (HBSS)**/** HEPES (Gibco, catalog no. 14170070, 1x; HEPES, Gibco, catalog no. 15630056, 10 mM) and centrifuged in a sterile 15 ml tube. The pellet was resuspended in 1 ml of HBSS/HEPES and dissociated through gentle pipetting. Fresh HBSS/HEPES (10 ml) was added, and the cells were centrifuged again. Finally, the pellet was resuspended in N2 medium (Dulbecco’s Modified Eagle Medium [DMEM]**/** F-12, Gibco, catalog no. 11320-074; N2 Supplement, 1x, Gibco, catalog no. 17502001; glucose, Merck Life Science, catalog no. G7021-100G, 3 g/L concentration; GlutaMAX, Thermo Scientific, catalog no. 35050087, 1x; apo-Transferrin, Merck Life Science, catalog no. T1428-500MG, 100 μg/ml concentration; NaHCO_3_, Merck Life Science, catalog no. S5761-500G, 3 mg/ml concentration; penicillin-streptomycin, Gibco, catalog no. 15140148, 100 U/ml concentration). Cells were inoculated at 1-1.5 × 10^6^ cells in 10 ml of N2 medium that was supplemented with 10 ng/ml bFGF (basic fibroblast growth factor, Alamone Labs, catalog no. F-170, 10 ng/ml concentration) per 10-cm dish coated with poly-L-ornithine (Merck Life Science, catalog no. P3655-50MG, 0.01% concentration in phosphate-buffered saline [PBS], Gibco, catalog no. 10010015), and fibronectin (Roche, catalog no. 10838039001, 5 µg/ml concentration in PBS). Fresh N2 medium was added to the cultures every other day. After 5 days, the cells were passaged to six-well plates and inoculated at 0.3 × 10^6^ cells per well in N2 medium that contained bFGF (10 ng/ml). After 24 h, fresh medium was added, supplemented with CNTF (Alamone Labs, catalog no. 1F-240, 20 ng/ml). Fresh CNTF was added every day.

### Induced pluripotent stem cell reprogramming and culture

Primary human fibroblasts were cultured in DMEM (Gibco, catalog no. 11965092) that was supplemented with 10% fetal bovine serum (Biowest, catalog no. fetal S181H-500) and penicillin-streptomycin (Gibco, catalog no. 15140148, 100 U/ml concentration) and maintained at 37°C in a 5% CO_2_ atmosphere_._ After the fourth cell passage, fibroblasts were split and seeded at a density of 1 × 10^4^ cells/cm^2^. After 24 h, in the presence of Polybrene Transfection Reagent (Merck Life Science, catalog no. TR-1003-G, 5 μg/ml), cells were transduced with a single lentiviral vector that expressed four transcription factors (Oct4, Klf4, Sox2, and cMyc) from a single transcript. After 48 h, transduced fibroblasts were split and cultured with a feeder layer of mitomycin C inactivated mouse embryonic fibroblasts (MEFs; Mitomycin C, Merck Life Science, catalog no. M5353, 10 μg/ml concentration) and cultured in iPSC medium (DMEM/F-12, Gibco, catalog no. 11320-074; 20% Knockout Serum Replacement, Gibco, catalog no. 10828010, 20% concentration; MEM Non-Essential Amino Acid Solution, Gibco, catalog no. 11140050, 1x; GlutaMAX, Thermo Scientific, catalog no. 35050087, 1x; penicillin-streptomycin, Gibco, catalog no. 15140148, 100 U/ml concentration; 2-mercaptoethanol, Gibco, catalog no. 31350010, 100 μM concentration; recombinant human bFGF protein, Alamone Labs, catalog no. F-170, 10 ng/ml concentration; sodium pyruvate, Gibco, catalog no. 11360070, 1 mM concentration). The iPSC medium was exchanged every other day for the next 3 weeks. After 21 days, iPSC colonies were manually collected, transferred to new fresh 5 cm Petri dishes, and cultured in iPSC medium. iPSCs were cultured for the next 20 days and passaged every 4 days. After this time, iPSCs were split and seeded on Matrigel-coated plates (Corning, catalog no. 356230) in Essential 8 Medium (Gibco, catalog no. A1517001). iPSCs were passaged every 4–5 days using Versene solution (Gibco, Cat No. 15040033) in the 24 h presence of the ROCK inhibitor (Tocris, catalog no. Y-27632, 10 μM concentration).

### Neural induction

iPSCs were seeded on Matrigel-coated plates in Essential 8 Medium that was supplemented with the ROCK inhibitor at a density of 2-2.5 × 10^5^ cells/cm^2^. The next day, the ROCK inhibitor was removed, and cells were cultured for the next 4 days in KOSR - Knockout Serum Replacement medium (DMEM/F-12, Gibco, catalog no. 11320-074; 20% Knockout Serum Replacement, Gibco, catalog no. 10828010, 20% concentration; MEM Non-Essential Amino Acid Solution, Gibco, catalog no. 11140050, 1x; GlutaMAX, Thermo Scientific, catalog no. 35050087, 1x; penicillin-streptomycin, Gibco, catalog no. 15140148, 100 U/ml concentration; 2-mercaptoethanol, Gibco, catalog no. 31350010, 100 μM concentration; LDN 193189 dihydrochloride [selective ALK2 and ALK3 inhibitor], Tocris, catalog no. 6053, 200 nM concentration; SB 431542 [selective TGF-βRI, ALK4, and ALK7 inhibitor], Tocris, catalog no. 1614, 10 nM concentration). After 5 days, cells were passaged, plated on Matrigel-coated plates, and cultured for the next 24 h in the presence of the ROCK inhibitor (SB 431542 and LDN 193189). After 24 h, fresh KOSR medium was added, supplemented only with LDN 193189, and cells were cultured for the next 96 h of culture. Afterward, the KOSR medium (100%) was replaced with mixture of 75% KOSR and 25% N2B27 medium (1:1 mixture of DMEM/F-12, Gibco, catalog no. 11320-074, and Neurobasal, Gibco, catalog no. 21103049, 1x; MEM Non-Essential Amino Acid Solution, Gibco, catalog no. 11140050, 1x; GlutaMAX, Thermo Scientific, catalog no. 35050087, 1x; penicillin-streptomycin, Gibco, catalog no. 15140148, 100 U/ml concentration; 2-mercaptoethanol, Gibco, catalog no. 31350010, 100 μM concentration; N2 Supplement, Gibco, catalog no. 17502001, 1x; B27, Gibco, catalog no. 17504001, 1x). For the next 3 days, cells were cultured with increasing concentrations of N2B27 in the mixture media (day 1, 50% N2B27, 50% KORS; day 2, 75% N2B27, 25% KORS; day 3, 100% N2B27, 0% KORS). After 12 days of culture, cells were passaged (1:2) and seeded on Matrigel-coated plates in NES medium (DMEM/F-12, Gibco, catalog no. 11320-074; GlutaMAX, Thermo Scientific, catalog no. 35050087, 1x; N2 Supplement, Gibco, catalog no. 17502001, 1x; B27, Gibco, catalog no. 17504001, 0.5x; penicillin-streptomycin, Gibco, catalog no. 15140148, 100 U/ml concentration; recombinant human bFGF protein, Alamone Labs, catalog no. F-170, 10 ng/ml concentration; recombinant human EGF, Alamone Labs, catalog no. E-100, 10 ng/ml concentration).

### Neural progenitor stem cell treatment

NPSCs were seeded in triplicate on Matrigel-coated plates in NES medium that was supplemented with the ROCK inhibitor. The next day, the ROCK inhibitor was washed out, and the medium was changed to differentiation medium (DMEM/F-12), Gibco, catalog no. 11320-074; GlutaMAX, Thermo Scientific, catalog no. 35050087, 1x; N2 Supplement, Gibco, catalog no. 17502001, 1x; B27, Gibco, catalog no. 17504001, 0.5x; penicillin-streptomycin, Gibco, catalog no. 15140148, 100 U/ml concentration, CNTF (Alamone Labs, catalog no. 1F-240, 20 ng/ml). Cells were cultured in a medium that was supplemented with CNTF for the next 5 weeks. Fresh medium that was supplemented with CNTF was added every third day. hNPSCs were routinely tested for mycoplasma. hNPSCs were not recently authenticated STR profiling.

### Brain organoids

Self-patterned whole-brain organoids from iPSCs were obtained according to the protocol of Lancaster et al. (2014) [64]. The organoids were cultured for 100 days.

### FACS purification of astrocytes

Cortices from *Aldh1l1*^eGFP^ embryos (E14.5 and E17.5) and pups (P7 and P35) or the somatosensory cortex from Control TdT and *Tcf7l2* cKO TdT mice (P35) were dissected and dissociated with papain (Worthington Biochemical, catalog no. LS003126, 20 U/ml concentration) for 70 min at 34 °C. Next, tissue was dissociated in Low Ovo Inhibitor Solution (EBSS [Earle′s Balanced Salt Solution], Merck Life Science, catalog no. E7510; glucose, Merck Life Science, catalog no. G7021-100G, 0.46% concentration; NaHCO_3_, Merck Life Science, catalog no. S5761-500G, 26 mM concentration; bovine serum albumin [BSA], Merck Life Science, catalog no. A8806, 1 mg/ml concentration; trypsin inhibitor, Merck Life Science, catalog no. 9253-1 G, 1 mg/ml concentration) and centrifuged for 10 min at 1200 rotations per minute, diluted in 1% BSA in PBS. Cell sorting was performed using BD FACS Aria II (BD Biosciences). Enhanced GFP (eGFP)-positive or TdT-positive cells were collected in post-sorting buffer (0.5% BSA, 10 mM HEPES, and 0.5% glucose [Merck Life Science, catalog no. G7021-100G]) and centrifuged at 500 × *g* for 10 min. The supernatant was removed, and the pellet was frozen at -80°C.

### RNA isolation and RNA-seq analysis

RNA from sorted cells (approximately 200,000 cells per sample) was isolated using the RNAeasy Mini Kit (Qiagen, catalog no. 74104, eGFP+ cells) or RNeasy Micro Kit (Qiagen, catalog no. 74004, TdT+ cells). The quality of RNA was verified using Bioanalyzer (Agilent). RNA samples from four Control TdT and three *Tcf7l2* cKO TdT animals were sequenced on the same run of Illumina HiSeq2500. Reads were aligned to the mouse genome mm10 assembly from the University of California, Santa Cruz (UCSC), using HISAT [65], and their counts were generated using HTSeq [66]. Differential gene expression analysis was performed with DeSeq2 [67]. Genes with a fold change ≥ 1.25 and < 0.75 and False Discovery Rate-adjusted *p* value (*q* value) ≤ 0.1 were considered differentially expressed up- and downregulated genes, respectively. The raw sequencing data are deposited in the EMBL Arrayexpress Annotare repository (https://www.ebi.ac.uk/fg/annotare/)

### Human tissue samples

De-identified human tissue samples were collected with prior patient consent in strict observance of legal and institutional ethical regulations. The protocols were approved by the Human Gamete, Embryo, and Stem Cell Research Committee (Institutional Review Board) at the University of California, San Francisco.

### Mouse brain fixation

Embryos were collected on E17.5. Timed-pregnant females were sacrificed by cervical dislocation. Embryos were removed and immediately decapitated. Brains were dissected, transferred to 4% paraformaldehyde (PFA; Sigma-Aldrich, catalog no. P6148), and fixed overnight in 0.1 M PBS (pH 7.4). Mice at P7 and older were anesthetized with a mixture of ketamine (Biowet, 125 mg/kg body weight) and xylazine (Biowet, 10 mg/kg body weight). Next, the mice were transcardially perfused with 0.1 M PBS, followed by 4.5% PFA (Merck Life Science, catalog no. P6148) in PBS. Brains were dissected, incubated overnight in 4.5% PFA, and saturated with 30% sucrose (Merck Life Science, catalog no. 1076515000) in PBS at 4 °C for 24 h. Next, the brains were transferred into O.C.T. (Sakura Tissue-Tek, catalog no. 4583) and frozen in -30°C isopentane (VWR, catalog no. 24872.298). Sections were obtained using a Leica CM1860 cryostat. Embryonic sections (10 μm) were mounted directly on SuperFrost-plus slides (Menzel-Gläser, catalog no. J1800AMNZ), and adult tissue sections (30 μm) were collected as free-floating sections in anti-freeze solution (30% sucrose, Merck Life Science, catalog no. 1076515000; 30% glycerol, VWR, catalog no. 443320113) 0.1 M PBS (pH 7.4).

### Immunocytochemistry

NSCs that were cultured on coverslips were fixed with 4.5% PFA in PBS, washed with PBS, blocked in 5% donkey serum (Merck Life Science, catalog no. S30-100ML) in PBST (PBS  +  0.2% Triton X-100, VWR, catalog no. 0694-1 L), and incubated overnight with primary antibodies (anti‐TCF7L2, Cell Signaling Technology, catalog no. 2569, 1:250 dilution; anti-SOX9, Biotechne, catalog no. AF3075-SP, 1:250 dilution; anti-GFAP, Merck Life Science, catalog no. Hpa056030, 1:250 dilution). The next day, the coverslips were washed and incubated with Alexa Fluor‐conjugated secondary antibodies (donkey anti-rabbit immunoglobulin G [IgG] with Alexa Fluor 488, Invitrogen, catalog no. A‐21206, 1:500 dilution; donkey anti-mouse IgG with Alexa Fluor 594, Invitrogen, catalog no. A‐21203, 1:500 dilution; donkey anti-mouse Author: IgG with Alexa Fluor 555, Invitrogen, catalog no. A-31570, 1:500 dilution; donkey anti-Goat IgG with DyLight 650, Invitrogen, catalog no. SA5-10089, 1:500 dilution). Finally, the coverslips were mounted on microscope slides with Fluoromount-G Mounting Medium with DAPI (Invitrogen, catalog no. 00-4959-52). Images were captured with an Axio Imager Z2 LSM 700 Zeiss confocal microscope.

### Immunohistochemistry

Frozen brain sections on slides or free‐floating sections were washed three times with PBST. Antigen retrieval was performed using sodium citrate buffer (Bioshop Life Science Products, catalog no. CIT001.1, 10 mM concentration; Tween 20, VWR, catalog no. 663684B, 0.05% concentration). Next, slices were blocked in 10% donkey serum in PBST and incubated overnight with primary antibodies (anti‐TCF7L2, Cell Signaling Technology, catalog no. 2569, 1:50 dilution; anti-SOX9, Biotechne, catalog no. AF3075-SP, 1:250 dilution; anti-β-catenin, Santa Cruz Biotechnology, catalog no. sc-7199, 1:100 dilution; anti-EGFP, Abcam, catalog no. ab13970, 1:1000 dilution; anti-Connexin-43, Invitrogen, catalog no. 13-8300, 1:200 dilution; anti-APC, Calbiochem, catalog no. OP80, 1:200 dilution; anti-Connexin-30, Invitrogen, catalog no. 71-2200, 1:200 dilution; anti-NeuN, Merck Millipore, catalog no. MAB377, 1:150 dilution; anti-OLIG2, Abcam, catalog no. ab9610, 1:200 dilution; anti-c-FOS Merck Millipore, catalog no. ABE457, 1:100 dilution; anti-VGAT, Synaptic System, catalog no. 131 004, 1:50 dilution; anti-gephyrin, Synaptic System, catalog no. 147 008, 1:50 dilution; anti-PSD-95, Invitrogen, catalog no. 51-6900, 1:50 dilution; anti-VGLUT1, Merck-Millipore, catalog no. AB5905, 1:50 dilution). The next day, the slices were washed and incubated with Alexa Fluor-conjugated secondary antibodies (listed above). Finally, slices were mounted on glass slides with Fluoromount G. Images were captured with an Axio Imager Z2 LSM 700 Zeiss confocal microscope.

### Western blot

Proteins from NSCs, hNPSCs, FACS-sorted astrocytes, or selected brain structures were extracted using ice‐cold RIPA buffer (Tris, Bioshop Life Science Products, catalog no. TRS001.1, 50 mM concentration, pH 7.5; NaCl, Chempur, catalog no. 794121116, 50 mM concentration; NP-40, Thermo Scientific, catalog no. 85124, 1% concentration; sodium deoxycholate, Merck Life Science, catalog no. D6750, 0.5% concentration; sodium dodecyl sulfate, Bioshop Life Science Products, catalog no. SDS999.500, 0.1% concentration; ethylenediaminetetraacetic acid [EDTA], VWR, catalog no. E177-500ML, 1 mM concentration; NaF, Merck Life Science, catalog no. 67414-1ML-F, 1 mM concentration; cOmplete, EDTA-free Protease Inhibitor, Roche, catalog no. 4693132001, 1x; phosphatase inhibitor, Roche, catalog no. 4906845001, 1x), centrifuged, and stored at -80°C. Proteins were separated in sodium dodecyl sulfate poly-acrylamide gels (Bio-Rad, catalog no. 1610183) and transferred to nitrocellulose membranes (Bio-Rad, catalog no. 1620112). Membranes were blocked with 10% nonfat dry milk in PBST (Tween 20, VWR, catalog no. 663684B, 0.1% concentration) and incubated overnight with primary antibodies (anti‐TCF7L2, Cell Signaling Technology, catalog no. 2569, 1:500 dilution; anti-GAPDH, Santa Cruz Biotechnology, catalog no. 25778, 1:500 dilution, anti-Vimentin, Abcam, catalog no. ab129002, 1:5000 dilution; anti-GFAP, Cell Signaling Technology, catalog no. 3670, 1:500 dilution) at 4°C. After washing, the membranes were incubated with secondary antibodies (anti-rabbit IgG peroxidase antibody, Sigma Aldrich, catalog no. A0545, 1:10000 dilution; anti‐mouse IgG peroxidase antibody, Sigma Aldrich, catalog no. A9044, 1:10000 dilution) for 2 h at room temperature. Staining was then visualized by chemiluminescence. Images were captured using an ImageQuant LAS 4000 (Cytiva). Densitometric analyses were performed using Quantity One 1‐D software (Bio-Rad).

### cDNA synthesis and RT-PCR

RNA from NSCs, hNPSCs or FACS-sorted astrocytes was transcribed to cDNA using the Transcriptor High Fidelity cDNA Synthesis Kit (Roche, catalog no. 05089284001). Transcript levels were measured using the SYBR Green I Master Kit (Roche, catalog no. 04887352001) and a LightCycler 480 Instrument II (Roche). Supplementary Table 2 contains a list of primers. The primers were designed using PrimerQuest. RNA from the cortex of *Aldh1l1*^eGFP^ transgenic mice was collected on E14, E17, P7 and P35. RNA was extracted using QIAzol (Qiagen, catalog no. 79306) and isolated using the RNAeasy Mini Kit (Qiagen, catalog no. 74104). The quality and purity of RNA were verified using nanodrop.

### Preparation of transfer plasmids for rAAV1/2 production

pZac2.1gfaABC1D-tdTomato plasmid (TdTomato plasmid) was purchased from Addgene. pZac2.1gfaABC1D-eGFP plasmid (eGFP plasmid) was constructed on a backbone of pZac2.1gfaABC1D-tdTomato. The TdTomato sequence was removed and replaced by the eGFP sequence. pAAV:ITR-U6-sgRNA(anti-*lacZ*)-gfaABC1D-Cre (control) and pAAV:ITR-U6-sgRNA-(anti-*Tcf7l2*)-gfaABC1D-Cre (*Tcf7l2* KO**)** transfer plasmids were constructed on a backbone of AAV:ITR-U6-sgRNA(backbone)-hSyn-Cre-2A-EGFP-KASH-WPRE-shortPA-ITR (Addgene, catalog no. 60231) [68], from which 2A-EGFP-KASH sequences were removed and the hSyn promoter was replaced by gfaABC1D that was cloned from pZac2.1 gfaABC1D-tdTomato (Addgene, catalog no. 44332) [69]. The pAAV:ITR-U6-sgRNA-hSyn-Cre transfer plasmid had only the 2A-EGFP-KASH sequence removed, allowing for guide efficiency tests on thalamic (TCF7L2-rich) neuronal cells. To construct the control plasmids, a guide sequence against *lacZ* (TGCGAATACGCCCACGCGAT) was cloned to be expressed in tandem with the gRNA scaffold under control of the hU6 promoter. For the experimental plasmids, the hU6-sgRNA cassette was duplicated, and a different guide sequence against critical exon 6 of the *Tcf7l2* gene (CGTCAGCTGGTAAGTGCGG; GGTGGGGGTGTTGCACCAC) was cloned into each. Guide sequences were designed with CHOPCHOP v3 Broad Institute GPP sgRNA Designer (https://portals.broadinstitute.org/gpp/public/analysis-tools/sgrna-design) and CRISPOR [69].

### Production, purification, and titration of rAAV1/2 particles

HEK293T cells in the exponential growth phase were simultaneously transfected with pAAV1 and pAAV2 rep/cap plasmids, pDF6 helper plasmid, and pAAV:ITR-U6-sgRNA-(anti-*Tcf7l2*)-gfaABC1D-Cre (*Tcf7l2* KO plasmid) for the generation of AAVs *Tcf7l2* KO, pAAV:ITR-U6-sgRNA-(anti-*lacZ*)-gfaABC1D-Cre (Control plasmid) for the generation of AAVs Control, pZac2.1gfaABC1D-tdTomato (TdT-plasmid) for the generation of AAVs TdT, and pZac2.1gfaABC1D-eGFP (eGFP plasmid) for the generation of AAVs GFP using PEI MAX MW 40,000 reagent (Polysciences, catalog no. 24765-1). pAAV1, pAAV2, and pDF6 plasmids were gifts from Lukasz Swiech (Broad Institute of MIT and Harvard, Cambridge, MA, USA). After 48–72 h, the cells were collected and lysed with sodium deoxycholate (Sigma-Aldrich, catalog no. D6750, concentration 0.5%). Free nucleic acids were digested with Benzonase Nuclease (Sigma-Aldrich, catalog no. catalog no. 70746, concentration: 50 units per ml), and leftover cellular components were discarded after centrifugation. The cell extract was then applied at a steady rate of 1 ml/min on a HiTrap Heparin HP Column (Cytiva, catalog no. 17040601), equilibrated with 150 mM NaCl and 20 mM Tris (pH 8.0) buffer. The column was then washed with 20 mM Tris buffer (pH 8.0) with increasing NaCl content (100, 200, and 300 mM), and viral particles were subsequently eluted using higher salt concentrations (400, 450, and 500 mM). Using Amicon Ultra-4 Centrifugal Filter NMWL 100 KDa (Millipore, catalog no. UFC810024), AAV particles were concentrated, and the buffer in which they were stored was exchanged for 1x PBS. The viral batch was sterilized by passing it through a Nanosep MF 0.2 µm centrifugal filter (Pall, catalog no. ODPTFE02C34), aliquoted, frozen in liquid nitrogen, and stored at -80°C. Titers of the AAV batch were verified by qPCR. Briefly, the AAV aliquot was treated with DNase (ThermoFisher Scientific, catalog no. AM2238), denatured at 95°C, and incubated with SmaI restriction enzyme (ThermoFisher Scientific, catalog no. FD0663) according to the manufacturer’s instructions. The numbers of DNA-containing viral particles were measured using the SYBR Green I Master Kit (Roche) and a LightCycler 480 Instrument II (Roche) in a series of the treated AAV dilutions using primers against the WPRE element. Series of transfer plasmid dilutions at known concentrations were used as references, and each sample was in triplicate. The viral batch was used for the experiments if its concentration was >1 × 10^9^ DNA-positive rAAV1/2 particles/µl.

### In vivo AAV1/2 injections

All stereotaxic injections were performed with a stereotaxic apparatus that was equipped with a microdispensing pump (Neurostar, catalog no. SD46) with a Nanofil syringe (World Precision Instruments, catalog no. 09 J, 10 μl volume). For perinatal injections of AAVs, P2/P3 animals of both sexes were anesthetized by hypothermia, and the head of the animal was fixed with a custom clay mold. Next, 0.5 µl of control AAVs or *Tcf7l2* KO AAVs (8 nl/s) was injected in the right ventricle using the following coordinates: anterior/posterior, 1.5 mm from lambda; medial/lateral, 0.9 mm; dorsal/ventral, -1.5 mm. After the procedures, the pups were placed on a 30°C heating pad and returned to their mother. The animals were sacrificed on P15. For perinatal injections of AAVs GFP and AAVs TdT, P2 animals were anesthetized by hypothermia and fixed with a custom clay mold. Next, 0.5 µl of AAVs GFP (8 nl/s) was injected in the right ventricle using the following coordinates: anterior/posterior, 1.5 mm from lambda; medial/lateral, 0.9 mm; dorsal/ventral, -1.5 mm. The next day, the animals were again anesthetized and fixed in a stereotaxic apparatus, and 0.5 µl of AAVs TdT (8 nl/s) was injected in the right ventricle using the following coordinates: anterior/posterior, 1.7 mm from lambda; medial/lateral, 0.9 mm; dorsal/ventral, –1.5 mm.

### Whole-cell patch-clamp recording

Thick slices (300 μm) were obtained from P30/P31 control and cKO mice. Slices were cut, recovered, and recorded in regular artificial cerebrospinal fluid that was composed of NaCl (Chempur, catalog no. 794121116, 119 mM concentration), KCl (Chempur, catalog no. 117397402, 2.5 mM concentration), MgSO_4_ (Sigma-Aldrich, catalog no. 1.06067, 1.3 mM concentration), CaCl_2_ (Chempur, catalog no. PA-07-12335, 2.5 mM concentration), NaH_2_PO_4_ (Sigma-Aldrich, catalog no. 74092, 1 mM concentration), NaHCO_3_ (Merck Life Science, catalog no. S5761-500G, 26.2 mM concentration), and glucose (Merck Life Science, catalog no. G7021-100G, 11 mM concentration), equilibrated with 95%/5% O_2_/CO_2_. The somata of layer 5a control and cKO neurons and astrocytes (*n* = 25 control neurons from 4 control mice; *n* = 44 cKO neurons from 8 Tcf7l2 cKO mice) in the somatosensory cortex were targeted for whole-cell patch-clamp recording with borosilicate glass electrodes (4-8 MΩ resistance). For neurons, the internal solution was composed of HEPES (Gibco, catalog no. 15630056, 10 mM concentration), EGTA (Sigma-Aldrich, catalog no. 324626, 0.5 mM concentration), MgATP (Sigma-Aldrich, catalog no. 9187, 4 mM concentration), NaGTP (Sigma-Aldrich, catalog no. G8877, 0.3 mM concentration), NaCl (Chempur, catalog no. 794121116, 8 mM concentration), cesium gluconate (HelloBio, catalog no. HB4822, 130 mM concentration), TEA-Cl (Sigma-Aldrich, catalog no. T2265, 10 mM concentration), and QX-314 (HelloBio, catalog no. HB1029), 5 mM concentration pH 7.25-7.35 (290 mOsm). Patch-clamp recordings were collected with a Multiclamp 700B amplifier (Molecular Devices), Digidata 1550 A digitizer, and pClamp 10.6 (Molecular Devices). Recordings were sampled at 20 kHz and filtered at 10 kHz. Analyses of mEPSCs and mIPSCs were performed using Clampfit 10.6.

### Gap junction assay

The gap junction assay was performed according to the protocol of Baldwin et al. (2021) [70]. Briefly, Neurobiotin Plus diffuses passively for 30 min in current-clamp mode. The slices were fixed in cold 4.5% paraformaldehyde in PBS overnight. The next day, the slices were washed with PBS that contained 0.5% Triton-X 100 and incubated in Streptavidin 488 at 1:500 (Life Technologies catalog no. S32354,) for 48 h at 4°C. Afterward, the slices were washed in PBST and mounted in homemade mounting media (90% glycerol, 20 mM Tris [pH 8.0], and 0.5% n-propyl gallate, Merck Life Science, catalog no. 02370). Z-stack confocal images from layer 5 of the somatosensory cortex of Control TdT and *Tcf7l2* cKO TdT were collected under a confocal microscope at a thickness of at least 40 μm. The quantification of neurobiotin-labeled cells was performed in ImageJ.

### Housing conditions and electronic tagging for behavioral tests in naturalistic settings

Animals were group-housed under a 12 h/12 h light/dark cycle with food and water provided *ad libitum*. Mice in the group tests were housed together in cohorts for at least 4 weeks before the experiment began to stabilize their social structure. Under brief isoflurane (5% GEULINCX) anesthesia, all mice were tagged by a subcutaneous injection of glass-coated microtransponders (11.5 mm length, 2.2 mm diameter, TSE Systems). Each microtransponder emits a unique identification code when the animal passes through RFID antennas or a portable chip reader. After the transponder installation procedure, the mice were moved from the housing facilities to the experimental rooms and adapted for 3 weeks to the reverse light/dark cycle (light phase, ZT 5; dark phase, ZT 17). In the housing and experimental rooms, the temperature was maintained at 23-24°C, with humidity between 35% and 45%.

### Cognitive assessment: reward learning

13 *Tcf7l2* cKO and 12 littermate control female mice were subjected to a 13-day protocol in two cohorts. The mice were first adapted to the experimental environment during the “simple adaptation” phase (3 days), “nose poke adaptation” phase (2 days), and “preparatory place learning” phase (3 days). At this time, all drinking bottles contained tap water. During “simple adaptation,” doors in all conditioning units (corners) were open, and access to water was unrestricted. During the following “nose poke adaptation,” all doors were closed by default and opened when an animal put its snout (nosepoke response) into one of the two holes on the operant learning chamber’s walls. The door remained open as long as the animal kept its snout in the hole, regardless of drinking behavior. During “preparatory place learning,” access to the drinking bottles was restricted to a single conditioning unit. A chamber with access to water was assigned randomly, with no more than three mice that drank from the same conditioning unit. Such a design has been shown to limit the social modulation of learning. This phase was followed by “reward learning” (5 days). The mice then had a choice between nosepoking for the bottle that contained tap water or the bottle that contained the reward (10% sucrose solution), which were placed behind two opposite doors in a conditioning chamber. Correct responses were defined as the percentage of nosepokes that were made for the bottle that contained the reward as the first choice after entering the conditioning unit. We also measured an increase in non-directed nosepoking (expressed as a percentage) after introduction of the reward into the experimental environment as a parameter that reflected difficulty in learning the reward location. For control purposes, we also recorded the total number of nosepokes and visits in the corners (activity).

### Behavior: in-cohort sociability

11 *Tcf7l2* cKO and 11 control female mice were subjected to a 72-h protocol of social behavior testing in two cohorts. The protocol was divided into an adaptation phase (24 h) and social behavior testing (72 h, testing in-cohort sociability and approach to social scent). During all phases of the experiment, the mice could freely explore all compartments of the naturalistic setting. In-cohort sociability was assessed during the 48 h testing period. For algorithms that allowed the calculation of in-cohort sociability, see Puscian et al. (2018) [44]. Code is available in an open source repository (https://github.com/Neuroinflab/pyEcoHAB). To test approach to the social scent, two novel olfactory stimuli were presented: social scent (bedding from the cage of an unfamiliar mouse of the same strain, sex, and age) and neutral scent (fresh bedding). Olfactory stimuli were placed behind the perforated partitions of the opposite testing compartments. Social approach was assessed as the ratio of approach to social *vs*. non-social odor during the first hour after introduction of the stimulus. The duration of the measurement was based on the activity level of the investigated mouse strain as previously described [41]. Social scent preference is a parameter that describes the time that an individual mouse spends near a social scent, calculated as the following: total time spent on day 4 (scents presented) in the Box C (T4_BoxC_) divided by total time spent in the Box A (T4_BoxA_); value 2 - total time spent on day 3 (no scents presented) in the Box C (T4_BoxC_) divided by total time spent in the Box A (T3_BoxA_);${{{{{\rm{a}}}}}}\,{{{{{\rm{social}}}}}}\; {{{{{\rm{scent}}}}}}\; {{{{{\rm{preference}}}}}}\; {{{{{\rm{parameter}}}}}}=\frac{{{{{{\rm{T}}}}}}{4}{{{{{\rm{BoxC}}}}}}{/}{{{{{\rm{T}}}}}}{4}{{{{{\rm{BoxA}}}}}}}{{{{{{\rm{T}}}}}}{3}{{{{{\rm{BoxC}}}}}}{/}{{{{{\rm{T}}}}}}{3}{{{{{\rm{BoxA}}}}}}}$; the following calculation was used to exclude a possible preference of mice to particular box

### Behavior: social novelty and social affiliation test in three-chambered apparatus

The social affiliation test was performed using a gray three-chambered apparatus (63 cm × 43 cm × 30 cm) that was divided into three compartments. Twenty-one control (12 females and 9 males) and 23 *Tcf7l2* cKO (12 females and 11 males) mice were habituated to the researcher and three-chambered apparatus for at least 1 week. Access to the side chambers (box A or C) was limited by a set of retractable doors. Testing was conducted in three consecutive sessions, each lasting 10 min. To test approach to social novelty, experimental mice were individually placed in the central chamber with access to the side chambers. After 10 min, the mouse was urged to return to the central chamber, and the doors were closed. In one of the chambers, a mouse 1 (stranger mouse 1 - unfamiliar same-sex C57Bl/6 J mouse of a similar age) was placed under the grid, and an inanimate object (wood block) was placed under the cup in the other chamber. The doors were then removed, and the experimental mouse was allowed to freely explore the arena. After 10 min, the mouse was again urged to return to the central chamber, and the doors were closed. The inanimate object was removed, and a novel mouse (stranger mouse 2 called social novelty unfamiliar same-sex C57Bl/6 J mouse of a similar age) was placed under the grid. The doors were then removed, and the experimental mouse was allowed to freely explore the arena. To test social affiliation, experimental mice were individually placed in the central chamber with access to the side chambers. After 10 min, the mouse was urged to return to the central chamber, and the doors were closed. In one of the chambers, a social scent (used bedding from the same-sex unfamiliar C57Bl/6 J mouse of a similar age) was placed under the grid, and a neutral scent (fresh unused bedding) was placed under the cup in the other chamber. The doors were then removed, and the experimental mouse was allowed to freely explore the arena. After 10 min, the mouse was again urged to return to the central chamber, and the doors were closed. Each test session was video recorded from above and analyzed offline using the automated EthoVision XT9 tool (Noldus). The following parameters were scored: distance traveled in each chamber, time spent in each chamber, number of visits to each chamber, distance traveled in close proximity to mouse 1or social novelty, time spent in close proximity to mouse 1or social novelty and number of visits in close proximity to mouse 1or social novelty.

### Exposure to social scent

Six control and nine *Tcf7l2* cKO mice were separated and transferred to new cages that contained fresh bedding. After 5 days of separation, each mouse was subjected to the olfactory stimulus protocol. An olfactory stimulus (social scent [used bedding from an unfamiliar same sex C57Bl/6 J mouse of a similar age] or neutral scent [fresh unused bedding]) was presented to the mouse. The social or neutral scent was placed in the middle of the cage. After 90 min, the mouse was anesthetized with ketamine (Biowet, 125 mg/kg body weight) and xylazine (Biowet, 10 mg/kg body weight) solution and transcardially perfused with 0.1 M PBS solution (pH 7.4), followed by 4.5% PFA in PBS.

### Quantification of astrocyte volume

The volume of astrocytes was analyzed using the MeasurementPro package in Imaris 8.4.2 software (Bitplane AG) by creating a three-dimensional surface rendering of individual astrocytes. Renders were created based on the pixel gradient intensity algorithm, and eGFP (488 nm channel) was used to masked individual astrocyte. Renders were thresholded to ensure all processes of astrocytes were properly reconstructed and maintained consistent thereafter. Pictures were blinded prior to analysis.

### Quantification of astrocyte tiling

Single-plane confocal pictures (nine from control mice, ten from *Tcf7l2* cKO mice) that contained two neighboring astrocytes (expressing either eGFP or TdTomato) were collected. The percentage of territory overlap between two neighboring astrocytes was calculated using the freehand selection tool in Fiji software. To measure the area of eGFP-positive astrocytes (green channel), TdT astrocytes (red channel) were removed, the border around the eGFP astrocytes was drawn using the freehand selection tool, and the area was calculated. Next, to measure the area of TdTomato-positive astrocytes (red channel), eGFP astrocytes (green channel) were removed, the border around TdT astrocytes was drawn using the freehand selection tool, and the area was calculated. Finally, overlapping territory was measured by drawing a border around the common area for eGFP and TdT astrocytes. Overlapping territory is shown as a percentage of the common area of two neighboring astrocytes.

### Quantification of synaptic puncta

The quantification of synaptic puncta was performed according to the protocol of Ippolito and Eroglu (2010) [42]. Briefly, pictures of synaptic puncta (z-stacks, 5 μm) from layer 5 of the somatosensory cortex (the same areas where mEPSCs and mIPSCs were analyzed) were collected under a confocal microscope with a 63 × objective. At least two z-stacks for each slice were collected. Pictures were blinded and analyzed using Fiji software. Positive puncta (white) were assessed manually by the experimenter based on the co-localization of pre- and postsynaptic markers (green and red). The data are expressed as an average for each mouse. At least two pictures from each mouse were collected.

### Quantification of connexin puncta

Single-plane confocal pictures from layer 5 of the somatosensory cortex of Control TdT and *Tcf7l2* cKO TdT mice were collected under a confocal microscope with a 63 × objective. The pictures were analyzed using ImageJ software. The number of Connexin puncta were automatically counted using the ImageJ Analyze Particles tool. The size of particles was thresholded for Control TdT and *Tcf7l2* cKO TdT pictures. The data are expressed as an average for each mouse. At least two pictures from each mouse were collected.

### Statistics, general methods and randomization

GraphPad Prism 9.5.1 software was used for statistical analyses. Data were analyzed using: two-tailed Mann–Whitney test, two-tailed unpaired *t* test with welch’s correction, one-way analysis of variance (ANOVA) or two-way ANAOVA, depending whether two or more groups were compared, to compare means, and two-sample Kolmogorov-Smirnov test to compare a cumulative distribution of data sets. For every figure, statistical tests were justified as appropriate and data met the assumptions of used tests. Individual data points are represented by dots in charts. The statistical tests are described in the figure legends. P  <  0.05 was considered significant. Outliers were excluded using Grubbs’ test. Sample size was chosen based on previous experience and published studies of the Authors. No statistical method was used to estimate the size of compared groups Randomization was not necessary. Blinding was done during quantification of synaptic puncta and volume of astrocytes.

### Supplementary information

List of differentially expressed genes from RNA-Seq

List of primers - RT-PCR

Extended Data Fig. 1

Extended Data Fig. 2

Extended Data Fig. 3

Extended Data Fig. 4

Extended Data Fig. 5

Extended Data Fig. 6

Extended Data Fig. 7

Extended Data Fig. 8

Extended Data Fig. 9

Extended Data Fig. 10

Extended Data Fig. 11

Extended Data Fig. 12

Extended Data Fig. 13

Extended Data Fig. 14

Extended Data Fig. 15

Extended Data Fig. 16

## Data Availability

The RNA-seq raw FASTQ files have been deposited in ArrayExpress under accession number E-MTAB-13153. All data are available in the main text or supplementary materials.
