# Supplementary figures and images for: Astrocytic β-catenin signaling via TCF7L2 regulates synapse development and social behavior
Source: Mol Psychiatry. 2023 Oct 5;29(1):57–73. doi: 10.1038/s41380-023-02281-y (PMC11078762; doi:10.1038/s41380-023-02281-y)

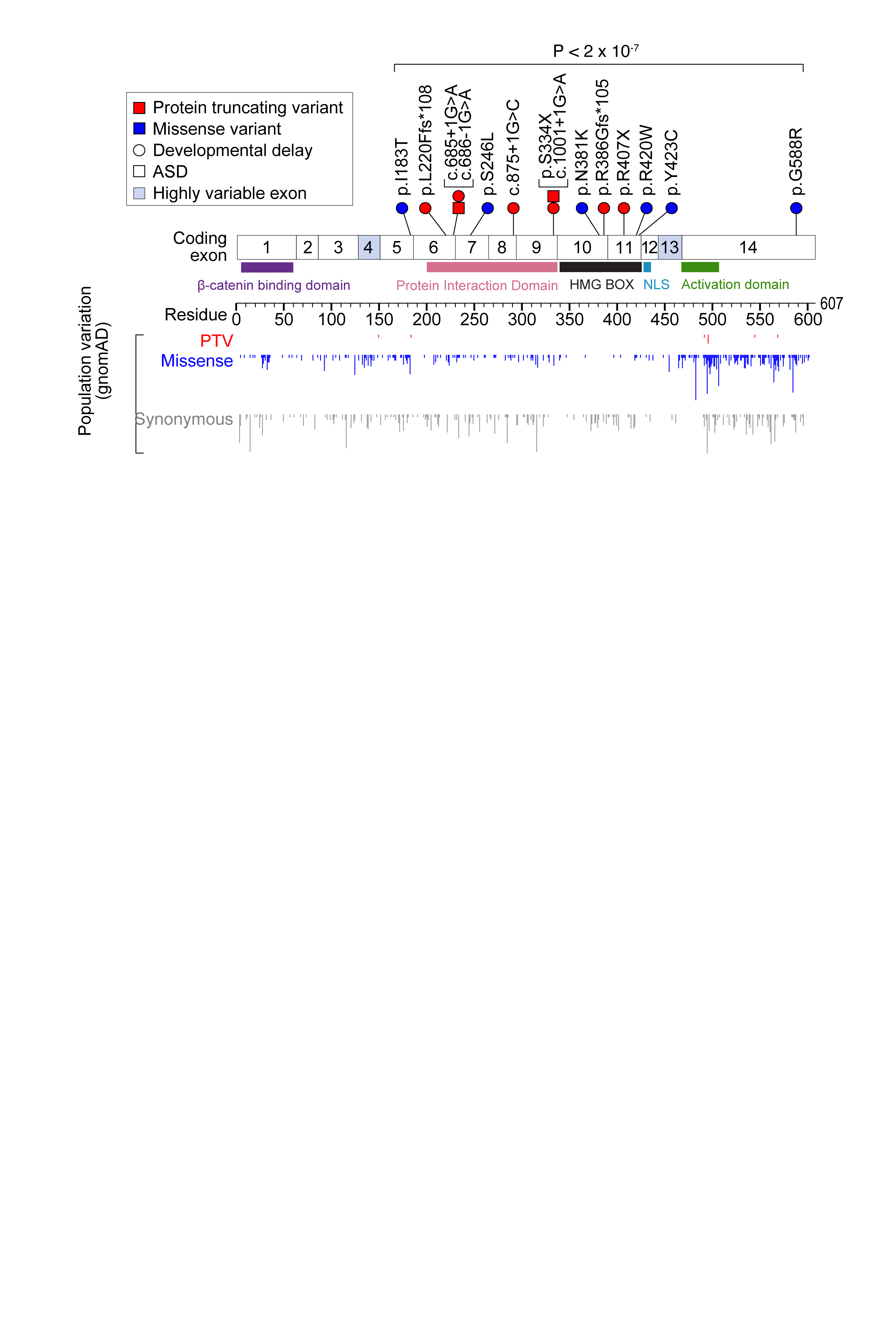

Supplement: Supplementary file 3 — Extended Data Fig. 1 [file 41380_2023_2281_MOESM3_ESM.tif]

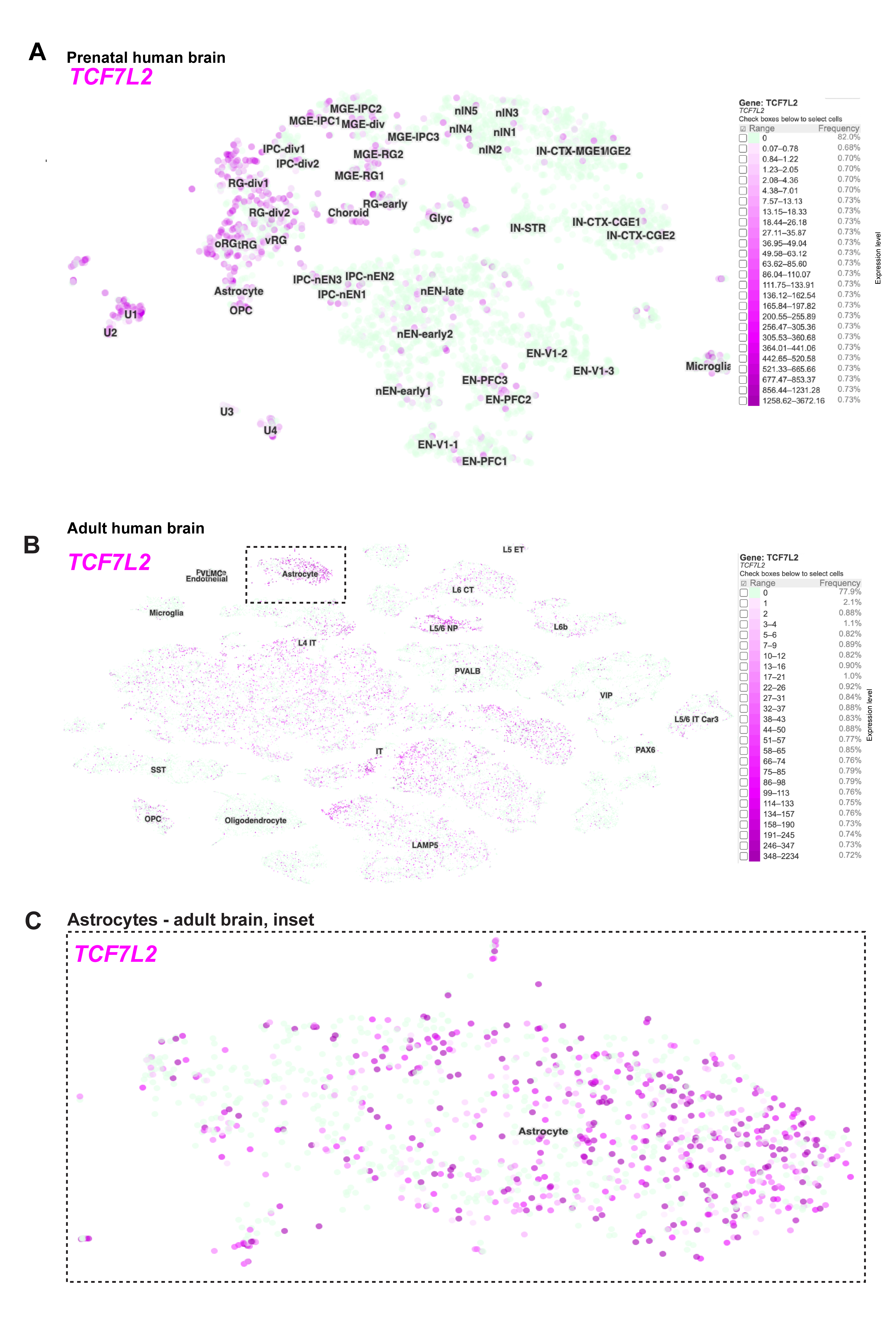

Supplement: Supplementary file 4 — Extended Data Fig. 2 [file 41380_2023_2281_MOESM4_ESM.tif]

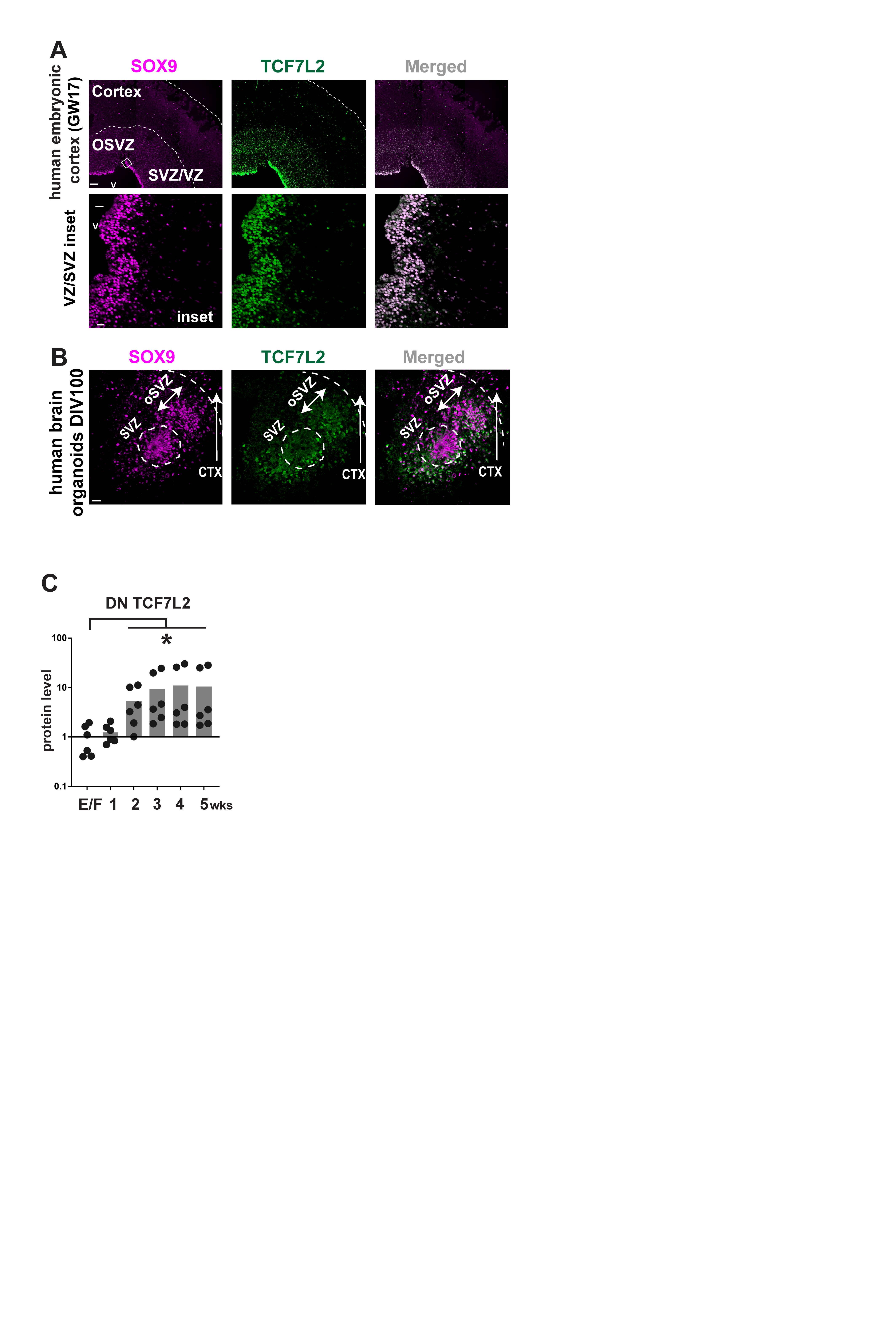

Supplement: Supplementary file 5 — Extended Data Fig. 3 [file 41380_2023_2281_MOESM5_ESM.tif]

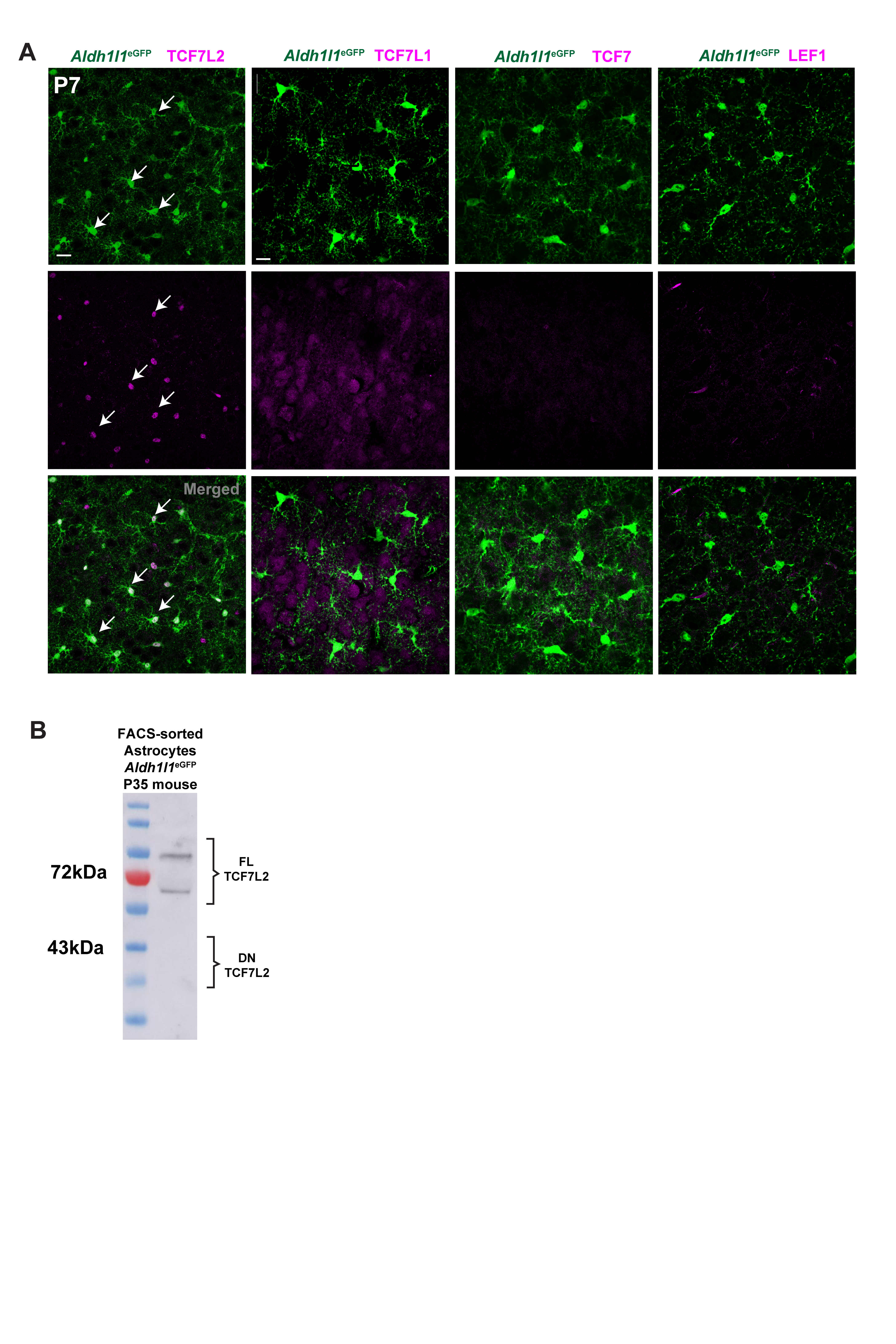

Supplement: Supplementary file 6 — Extended Data Fig. 4 [file 41380_2023_2281_MOESM6_ESM.tif]

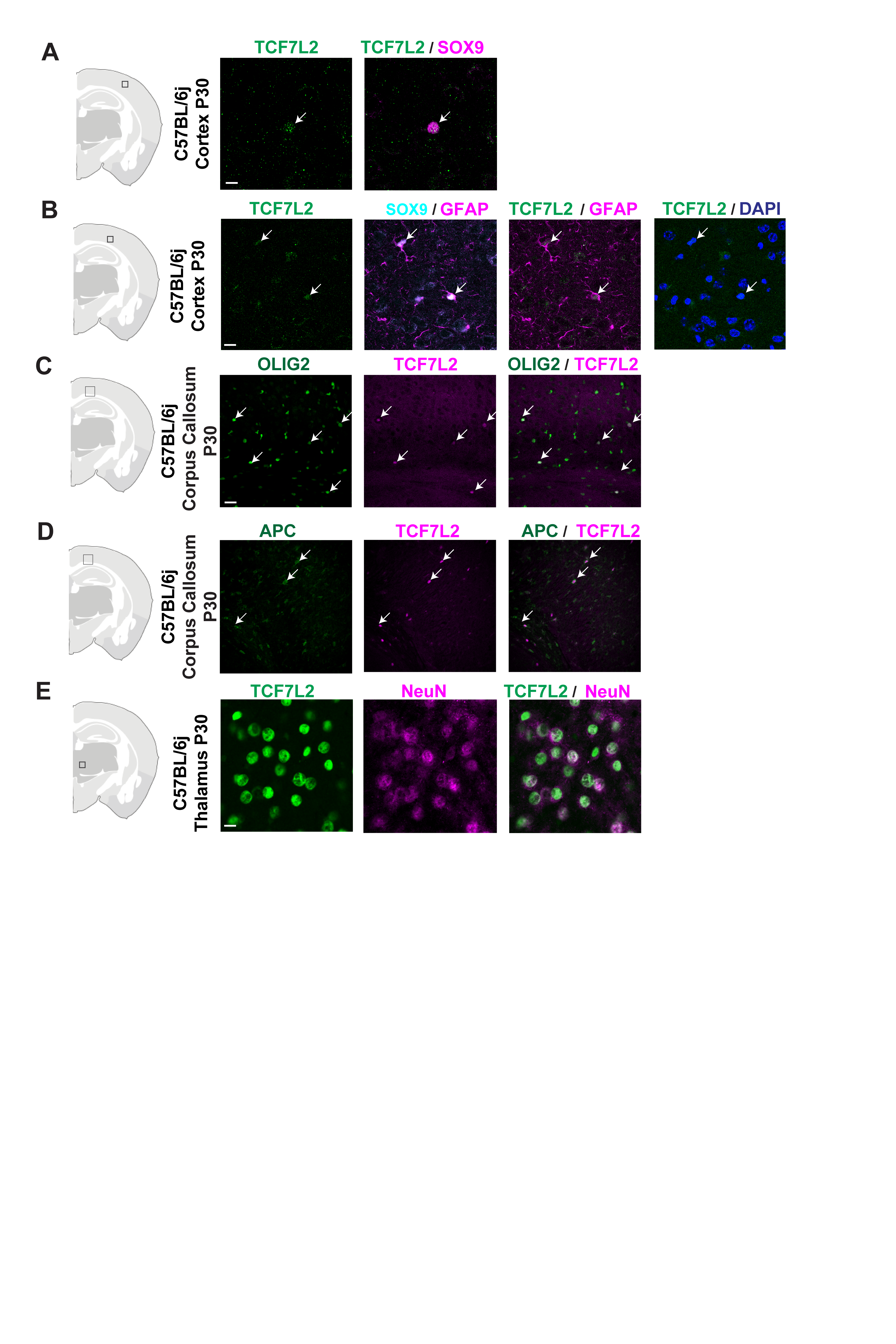

Supplement: Supplementary file 7 — Extended Data Fig. 5 [file 41380_2023_2281_MOESM7_ESM.tif]

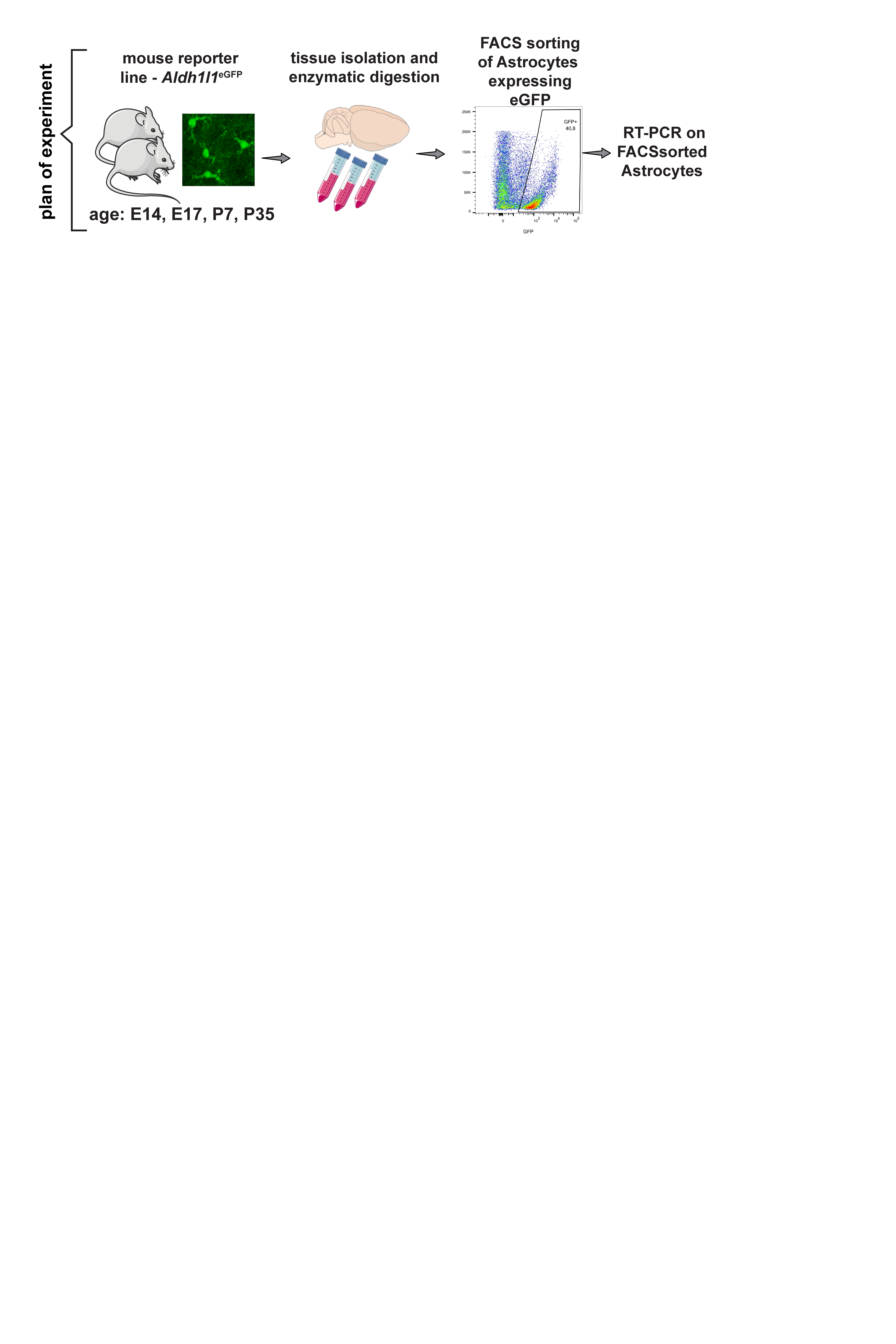

Supplement: Supplementary file 8 — Extended Data Fig. 6 [file 41380_2023_2281_MOESM8_ESM.tif]

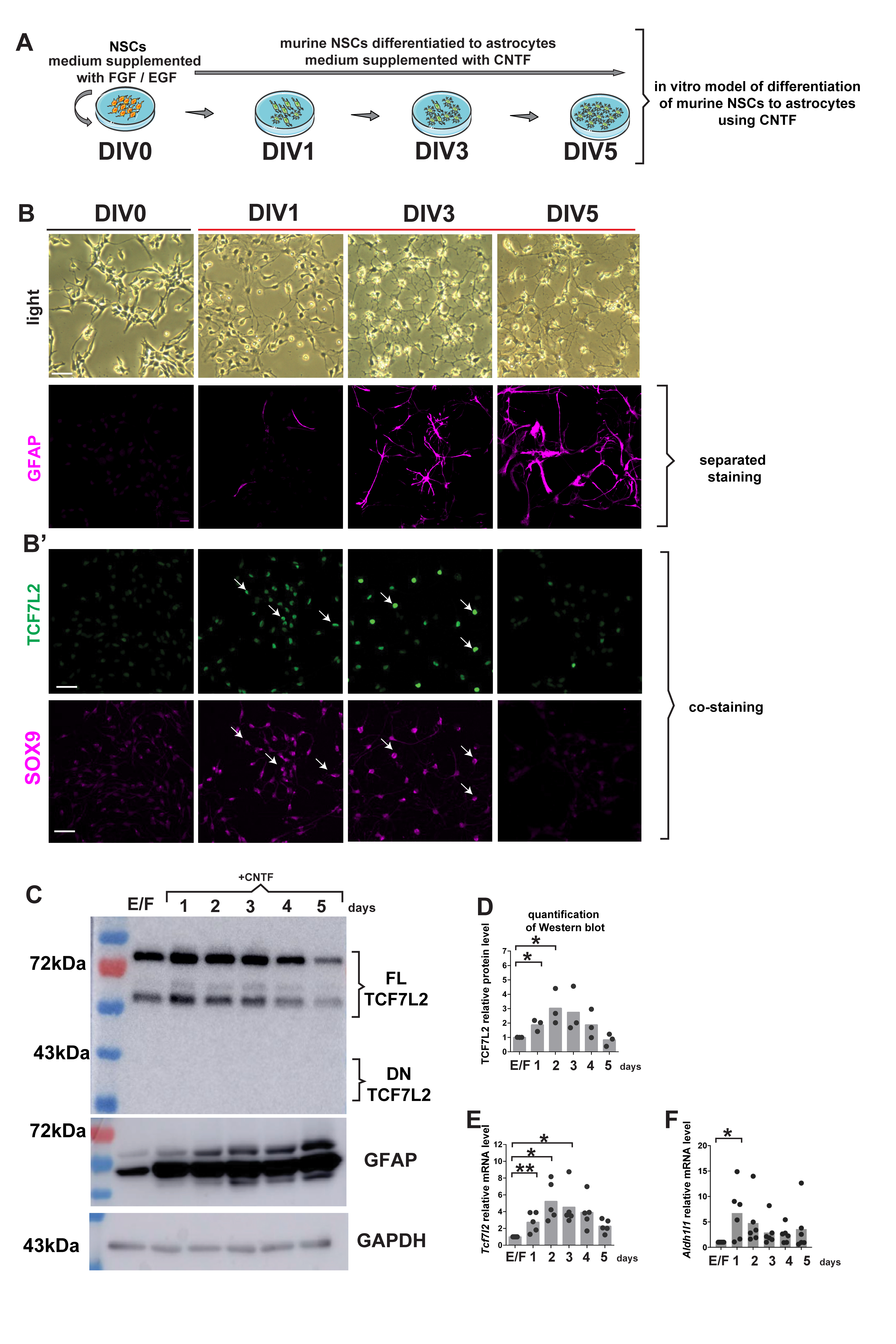

Supplement: Supplementary file 9 — Extended Data Fig. 7 [file 41380_2023_2281_MOESM9_ESM.tif]

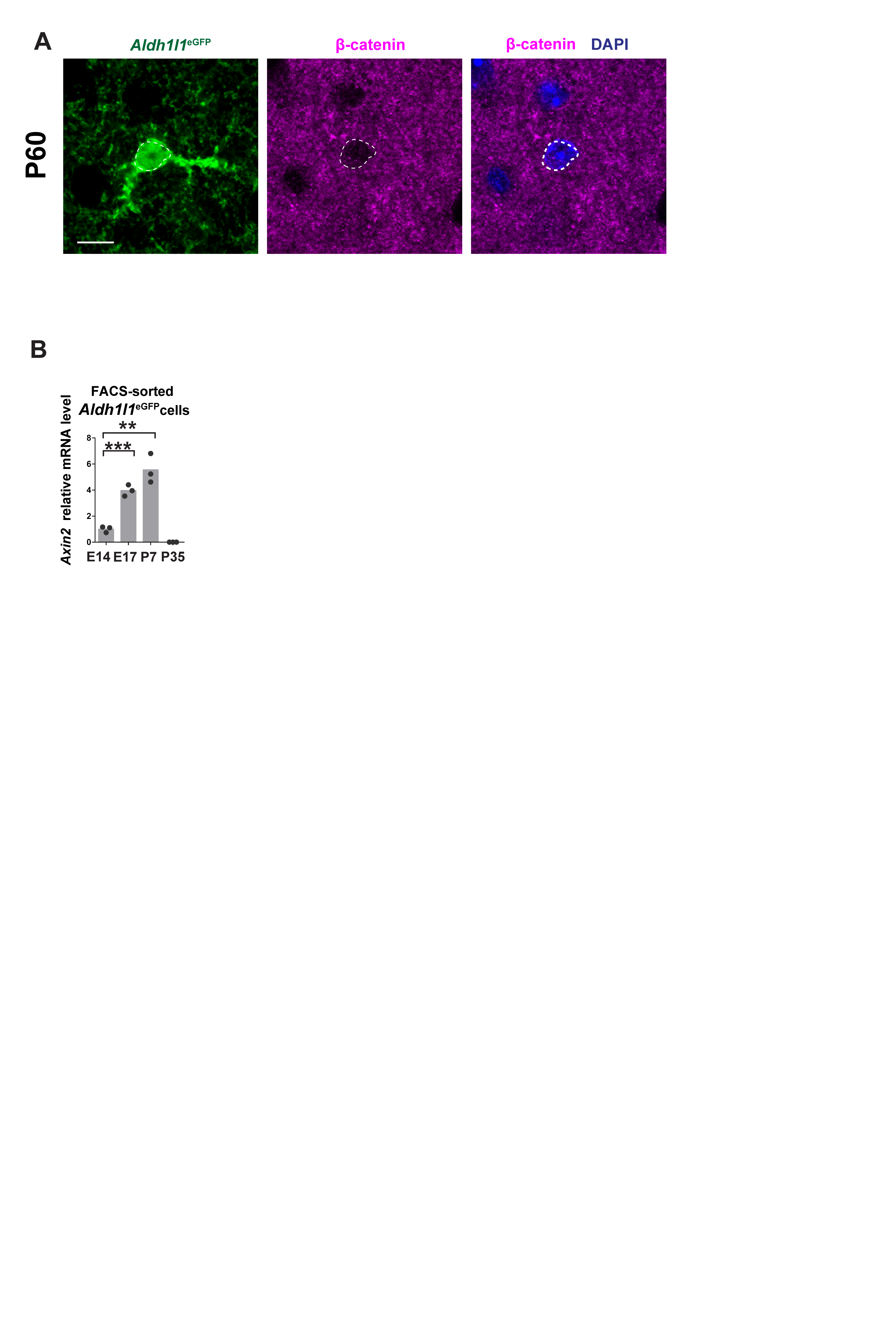

Supplement: Supplementary file 10 — Extended Data Fig. 8 [file 41380_2023_2281_MOESM10_ESM.tif]

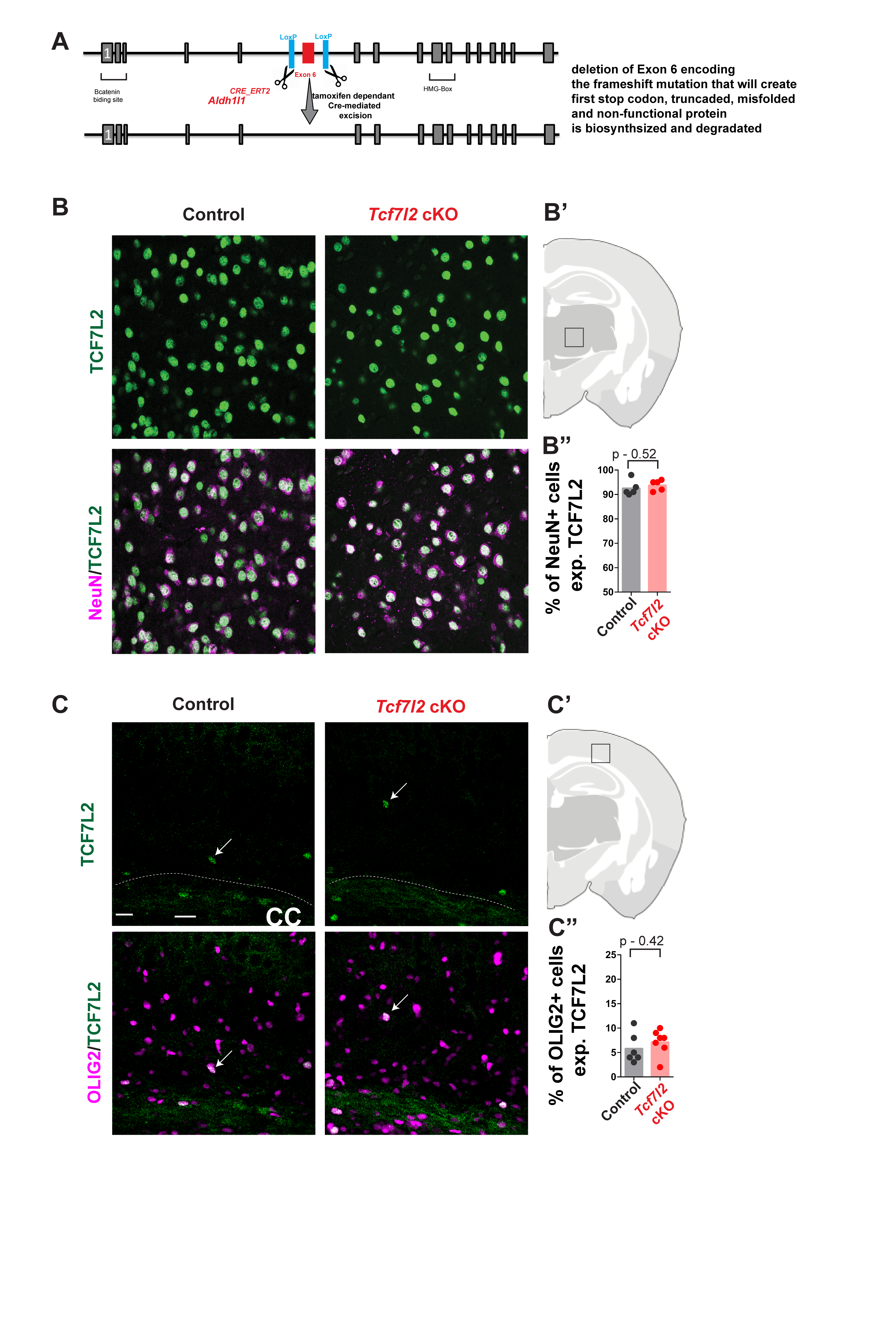

Supplement: Supplementary file 11 — Extended Data Fig. 9 [file 41380_2023_2281_MOESM11_ESM.tif]

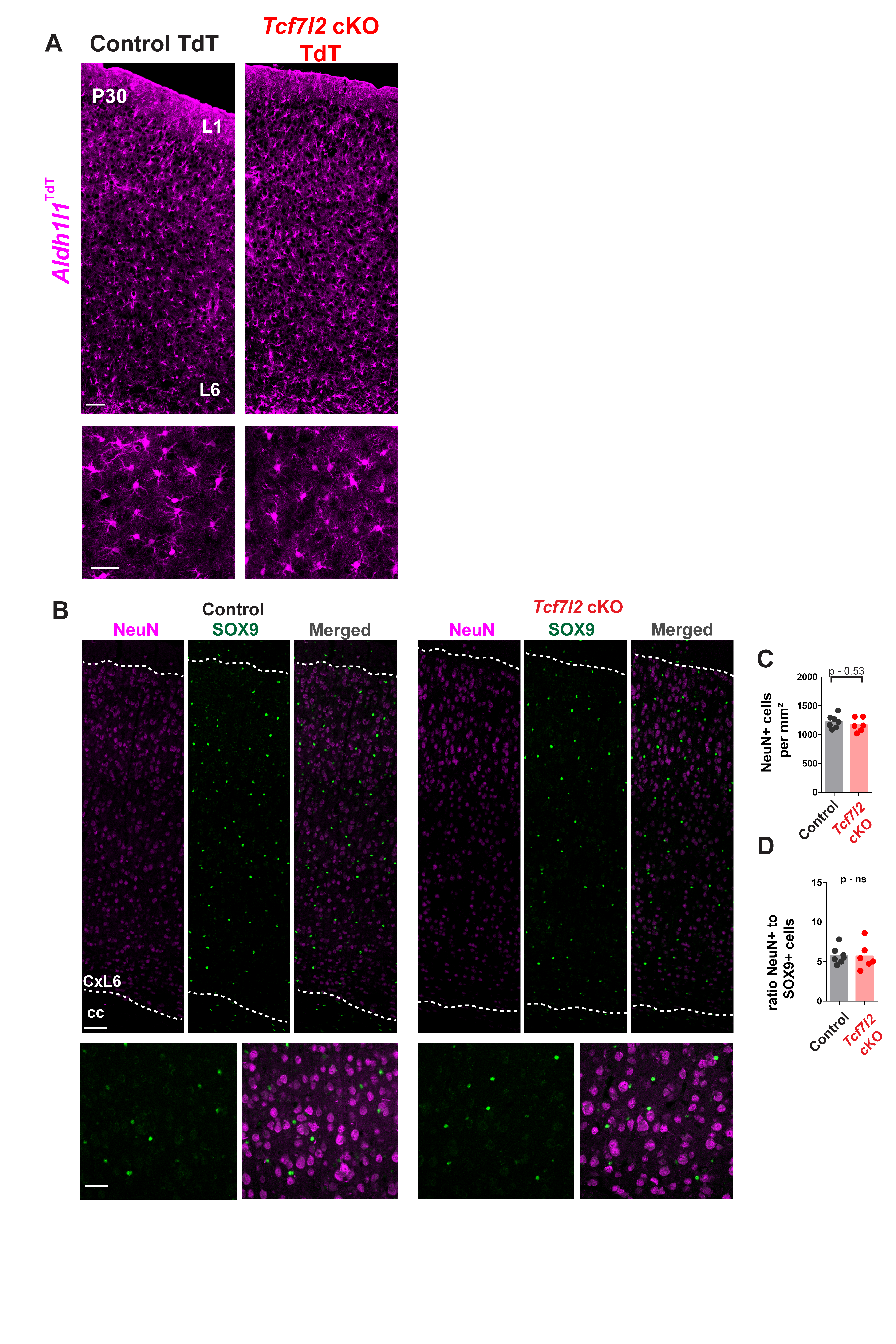

Supplement: Supplementary file 12 — Extended Data Fig. 10 [file 41380_2023_2281_MOESM12_ESM.tif]

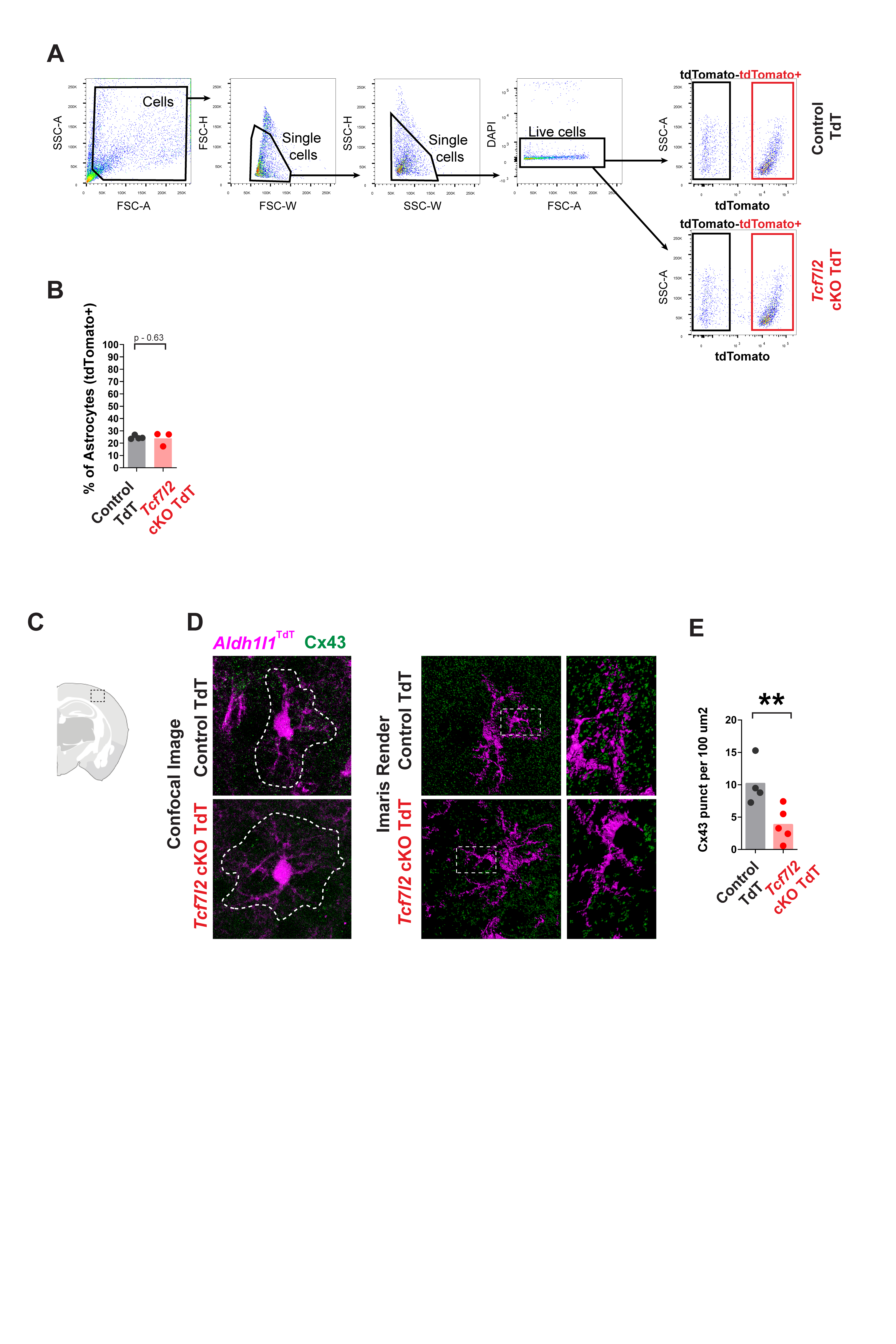

Supplement: Supplementary file 13 — Extended Data Fig. 11 [file 41380_2023_2281_MOESM13_ESM.tif]

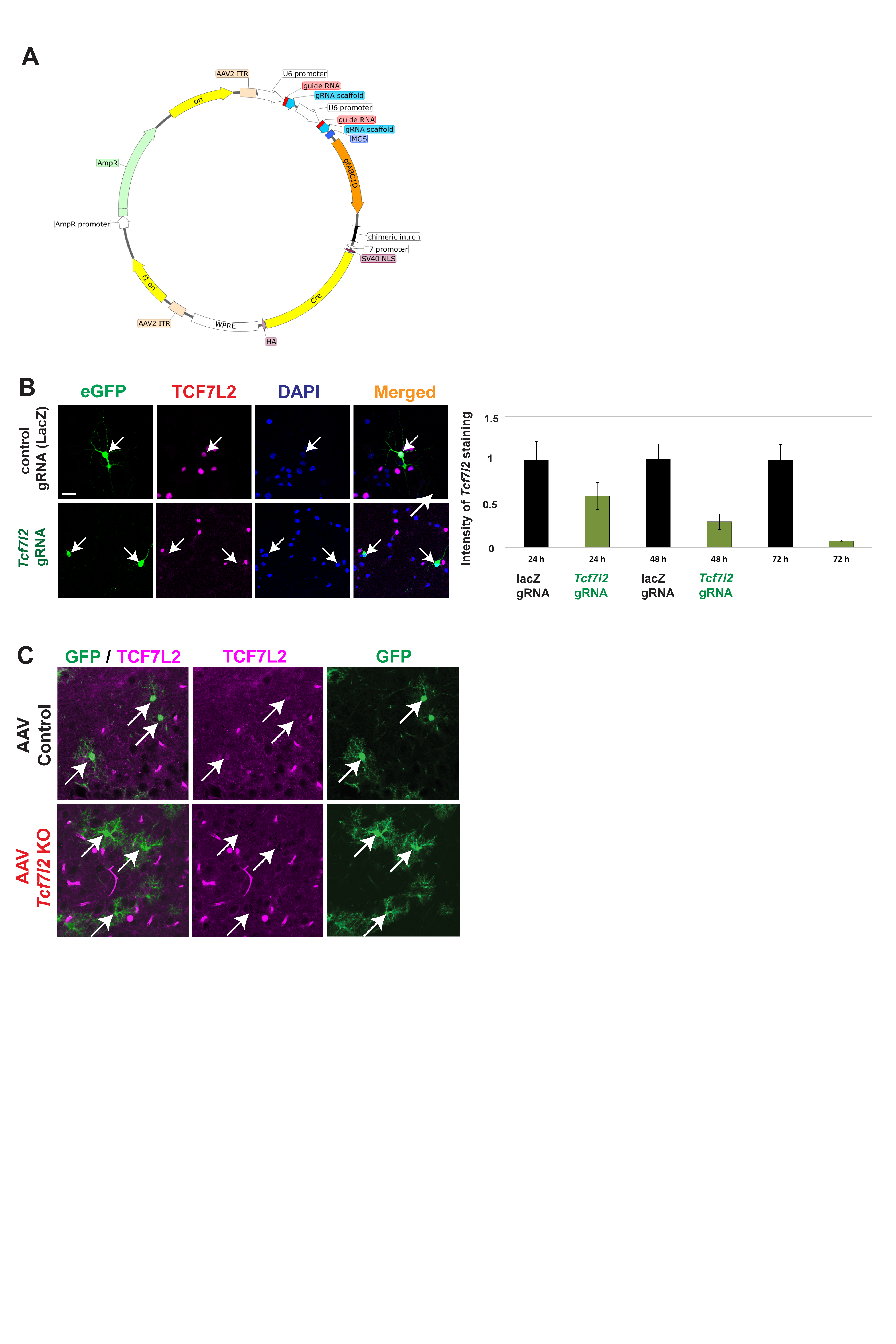

Supplement: Supplementary file 14 — Extended Data Fig. 12 [file 41380_2023_2281_MOESM14_ESM.tif]

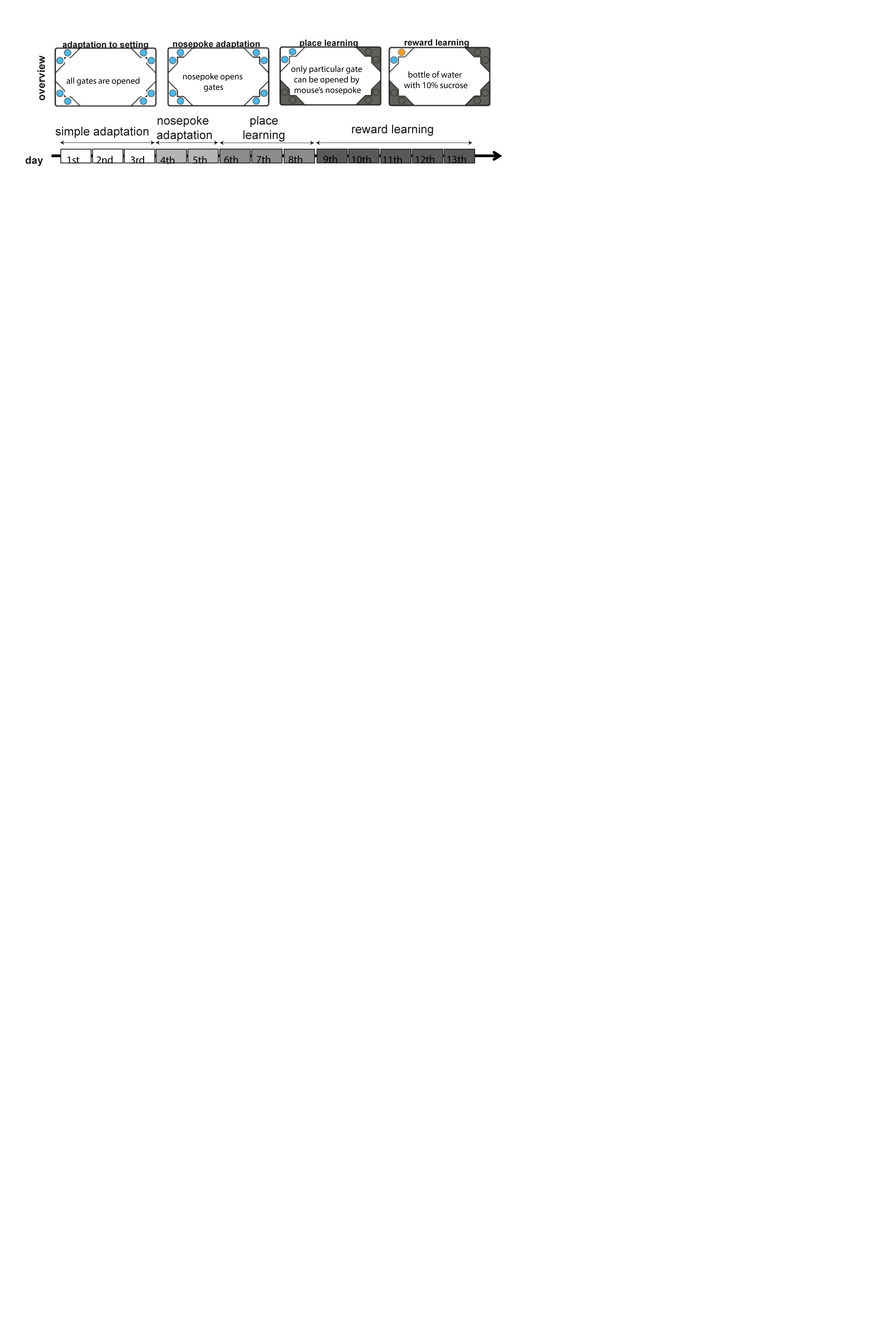

Supplement: Supplementary file 15 — Extended Data Fig. 13 [file 41380_2023_2281_MOESM15_ESM.tif]

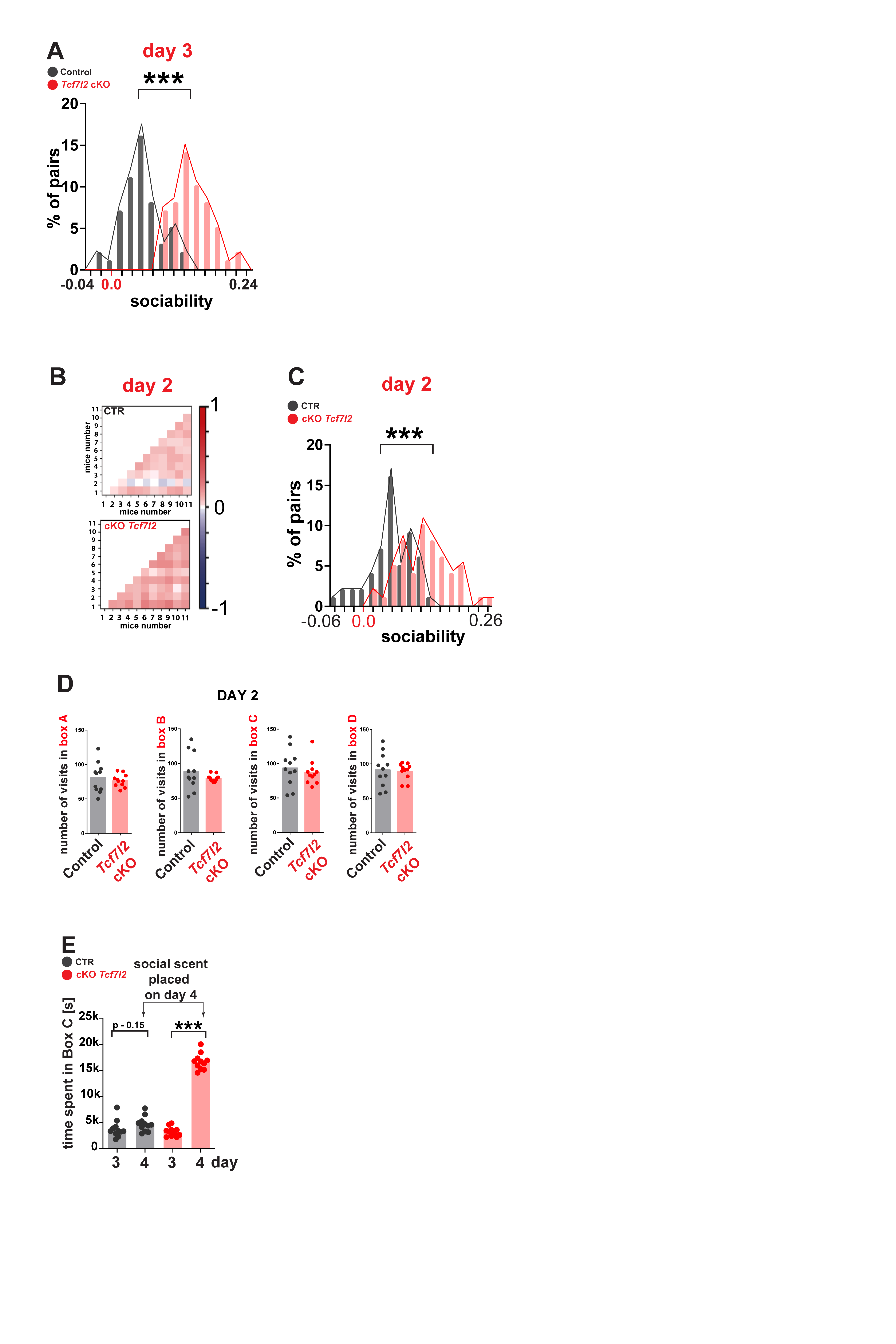

Supplement: Supplementary file 16 — Extended Data Fig. 14 [file 41380_2023_2281_MOESM16_ESM.tif]

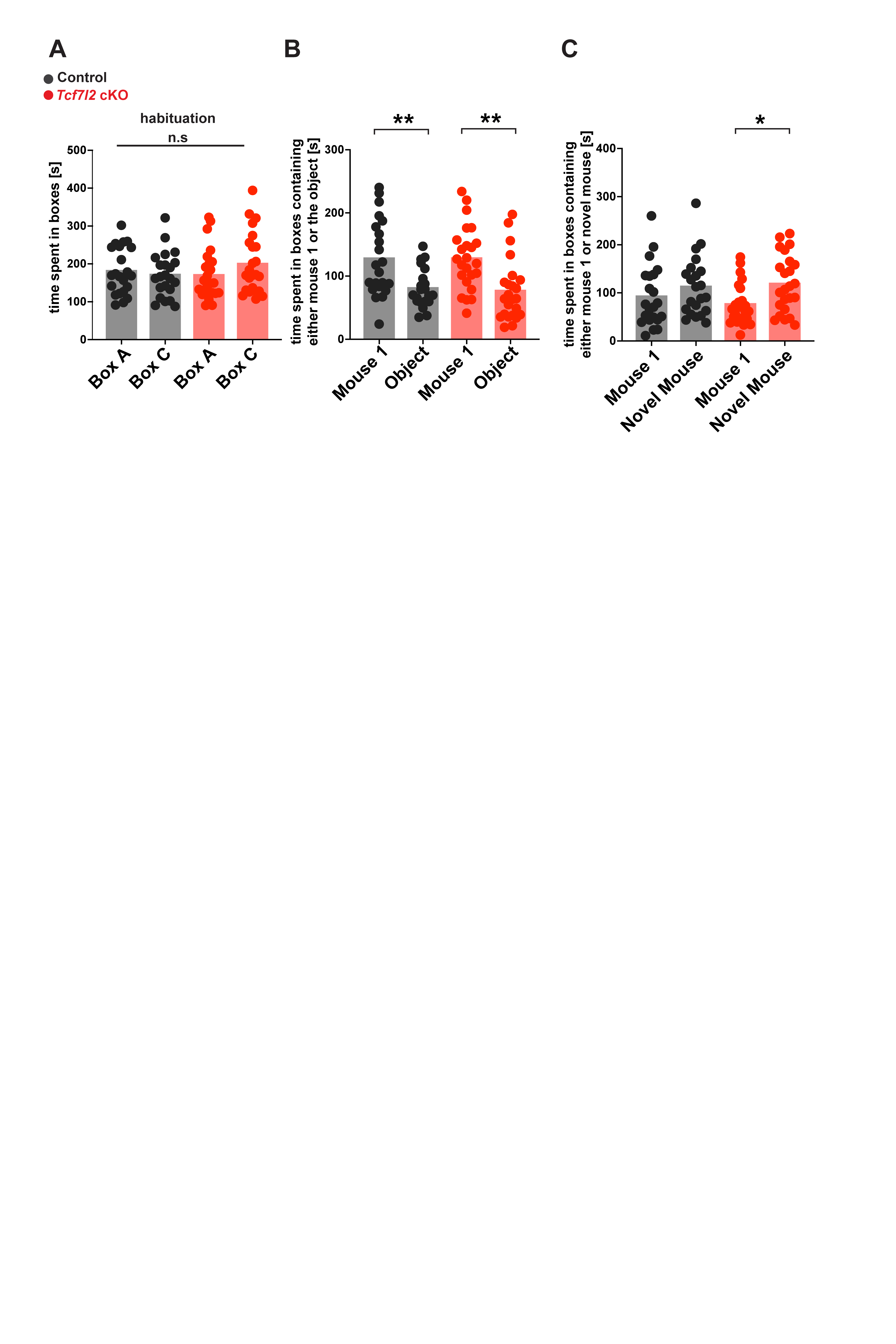

Supplement: Supplementary file 17 — Extended Data Fig. 15 [file 41380_2023_2281_MOESM17_ESM.tif]

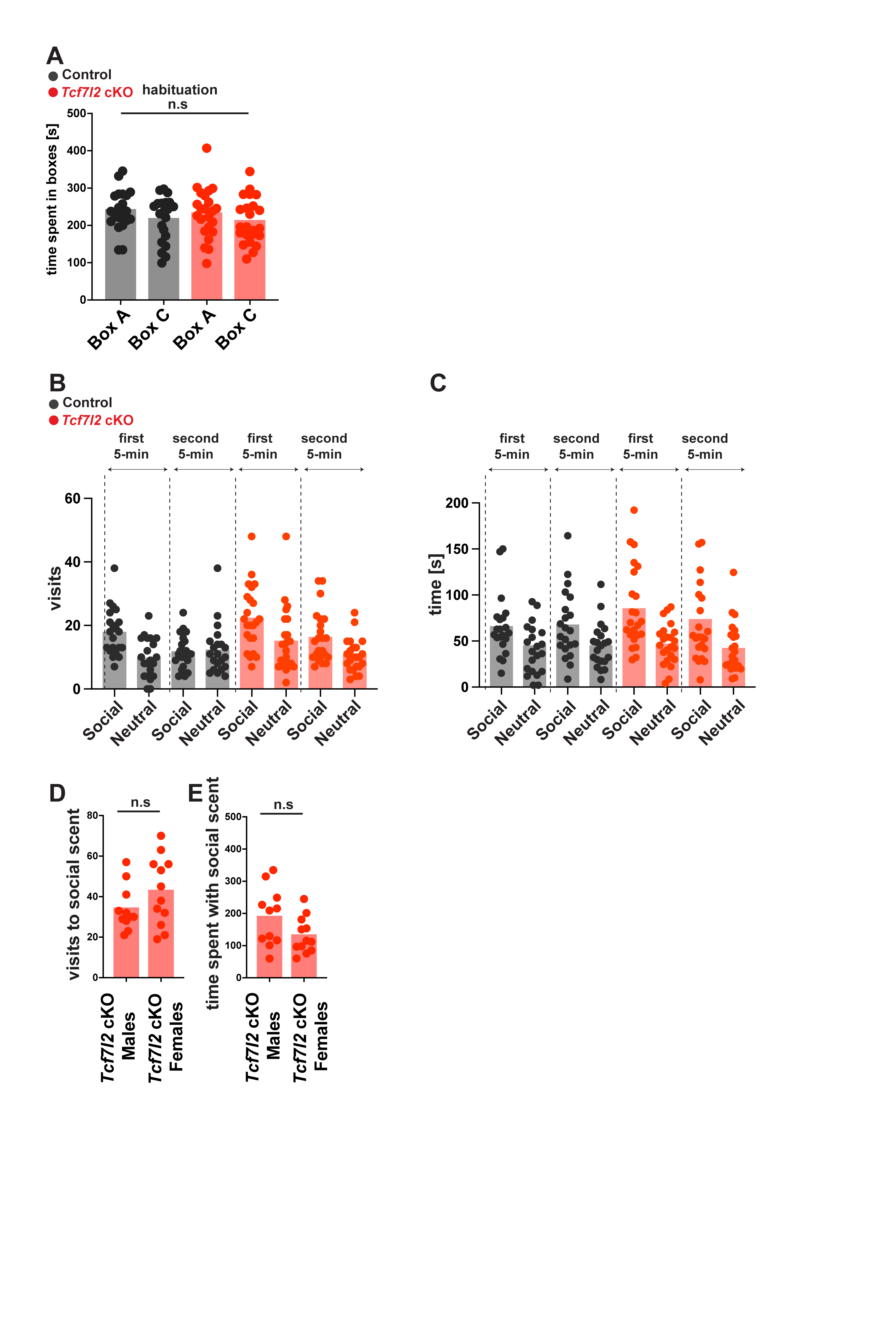

Supplement: Supplementary file 18 — Extended Data Fig. 16 [file 41380_2023_2281_MOESM18_ESM.tif]
